# Elevated concentrations cause upright alpha-synuclein conformation at lipid interfaces

**DOI:** 10.1038/s41467-023-39843-1

**Published:** 2023-09-18

**Authors:** Steven J. Roeters, Kris Strunge, Kasper B. Pedersen, Thaddeus W. Golbek, Mikkel Bregnhøj, Yuge Zhang, Yin Wang, Mingdong Dong, Janni Nielsen, Daniel E. Otzen, Birgit Schiøtt, Tobias Weidner

**Affiliations:** 1 Department of Chemistry, Aarhus University, Langelandsgade 140, 8000 Aarhus C, Denmark; 2 Department of Anatomy and Neurosciences, Amsterdam UMC, Vrije Universiteit, De Boelelaan 1108, 1081 HZ Amsterdam, The Netherlands; 3 Interdisciplinary Nanoscience Center (iNANO), Aarhus University, Gustav Wieds Vej 14, 8000 Aarhus C, Denmark

**Keywords:** Proteins, Parkinson's disease, Ultrafast photonics, Supramolecular assembly, Computational biophysics

## Abstract

The amyloid aggregation of *α*-synuclein (*α*S), related to Parkinson’s disease, can be catalyzed by lipid membranes. Despite the importance of lipid surfaces, the 3D-structure and orientation of lipid-bound *α*S is still not known in detail. Here, we report interface-specific vibrational sum-frequency generation (VSFG) experiments that reveal how monomeric *α*S binds to an anionic lipid interface over a large range of *α*S-lipid ratios. To interpret the experimental data, we present a frame-selection method ("*ViscaSelect*”) in which out-of-equilibrium molecular dynamics simulations are used to generate structural hypotheses that are compared to experimental amide-I spectra via excitonic spectral calculations. At low and physiological *α*S concentrations, we derive flat-lying helical structures as previously reported. However, at elevated and potentially disease-related concentrations, a transition to interface-protruding *α*S structures occurs. Such an upright conformation promotes lateral interactions between *α*S monomers and may explain how lipid membranes catalyze the formation of *α*S amyloids at elevated protein concentrations.

## Abstract (web summary)

The misfolding of alpha-synuclein is associated with neurodegenerative disorders such as Parkinson’s disease. The authors report a mechanism explaining why lipid membranes catalyze the formation of harmful aggregates at elevated concentrations.

## Introduction

Amyloid aggregation of misfolded proteins is associated with a large variety of diseases^1–3^. Aggregation of *α*-synuclein (*α*S) is related to serious and incurable *α*-synucleinopathies such as Parkinson’s disease^4–7^. *α*S is a 140-residue protein with an N-terminal region (residues ∼1–60) that contains several positively charged residues and a highly negatively charged C-terminal region (residues ∼96–140). The non-amyloid *β*-component (NAC) region, residue ∼61–95, forms the hydrophobic core in *α*S fibrils and is thus implicated in *α*S aggregation^8^. Lipid interfaces are thought to play an important role in the aggregation of *α*S^9,10^. The relevance of the aggregation catalysis by lipids is substantiated by the presence of lipids in Lewy bodies, the neuronal inclusions that are a pathological hallmark of Parkinson’s disease^11–13^ and by the observation of lipid–protein co-aggregates in vitro^12,14,15^. Obtaining a molecular understanding of the lipid-*α*S interaction could be of vital importance for resolving the contribution of *α*S aggregation to Parkinson’s disease, as anionic lipid membranes are known to catalyze *α*S amyloid formation^14,16^. The interaction between *α*S and lipid membranes has therefore been extensively studied and reviewed^9,10,17^. Structural studies of *α*S at membrane interfaces have mainly been based on nuclear magnetic resonance (NMR)^18–23^, electron paramagnetic resonance (EPR)^18,21^ and neutron reflectivity (NR)^24^ experiments, with *α*S bound to lipid vesicles^19,20,22^, surfactant micelles^18,19,21,23^, and lipid bilayers^19,24^.

The density of *α*S at the membrane surface, depending on the *α*S concentration in solution relative to the number of lipids, is known to be an important factor for the aggregation^16,25–27^. The relative concentration of *α*S is often expressed as the lipid–protein ratio (LPR), which denotes the molar ratio between lipid and protein, i.e., a low LPR denotes a high relative *α*S concentration. In studies with lipid monolayers, the LPR can be calculated using the known concentration of added protein, the volume of the solution, and the area per lipid at the interface. It has been shown that below an LPR of 10, *α*S clusters form that induce membrane damage due to amyloid formation^28^. NMR, EPR, and NR studies provide a molecular-level image of *α*S interaction but have mainly been limited to relatively high LPRs (i.e., relatively low *α*S concentrations)^10,17,23^. In the literature, there is only little structural information available at the molecular level for lower LPRs, i.e., higher protein concentrations, where *α*S aggregates into amyloids catalyzed by the lipid interface (starting at ∼10 μM for supported lipid bilayers^28^, while the physiological concentration of *α*S is ∼20 μM^29^). This lack of knowledge may partly be explained by the difficulty of performing NMR measurements to study protein structure at interfaces in the presence of a large number of proteins in the bulk phase. Since *α*S has a relatively weak affinity to most lipids^16,20,30^, forming densely packed protein layers at the lipid interface requires high solution concentrations. Such *α*S concentrations are of particular interest to study, given the strong correlation that has been observed between elevated *α*S concentrations and aggregation-related toxicity^31^.

One particularly suited technique to study surface-bound proteins in the presence of high bulk concentrations is vibrational sum-frequency generation (VSFG)^32,33^. VSFG signals are, per selection rules, only generated at interfaces. Only surface-interacting proteins contribute to the observed signal, while background signals from proteins in bulk do not contribute. To provide structural insights into *α*S at low concentrations as applied before in the literature, but also at physiological and elevated concentrations, we have performed VSFG amide-I spectroscopy of *α*S at an anionic lipid monolayer interface. In combination with molecular dynamics (MD) simulations, we obtain a molecular picture of lipid-associated *α*S structure when bound to an anionic 1,2-dipalmitoyl-${sn}$-glycero-3-phosphoglycerol (DPPG) monolayer interface at the physiological ionic strength and pH. While a cellular membrane has a variety of lipids carrying different charges, we choose a uniform DPPG monolayer as a model system so that we can focus on the effect of varying *α*S concentration near negatively charged membranes.

In recent years, VSFG has matured into a technique that is frequently employed to study proteins at interfaces^32,33^. Previously, the combination of VSFG with MD simulations has enabled researchers to interpret protein VSFG data at a molecular level^34–38^. In the present study, we have extended this approach. MD simulations are used to generate a large number of hypothetical *α*S conformations in a lipid-bound state, which is subsequently evaluated with spectral calculations for each snapshot or ‘frame’ (i.e., conformation) of the MD trajectories. This frame-selection method (using the “*ViscaSelect*” algorithm, a part of the *Visca* vibrational spectroscopy calculation tools, see the *Code availability* section) thus results in an ensemble of conformations that agrees best with the VSFG experiments, which can be structurally inspected with further analyses.

With this methodology, we derive that *α*S undergoes a conformational transition from a flat to an upright conformation as a function of increasing local concentration. The VSFG spectra that we recorded at relatively low concentrations (50 nM to 20 μM, equaling LPRs of ∼37 to 0.1) are consistent with the flat-lying *α*-helical structures that have been observed before^18–24^. However, at an elevated concentration of ∼50 μM (LPR = ∼0.04, equaling ∼2.5 times the physiological concentration^29^), the protein adopts an elongated structure with intermediate helicity, which is bound with its first ∼50 residues to the lipid interface, while the rest of the protein is generally oriented upright (i.e., perpendicular) with respect to the lipid surface. Such structures have not been reported in the literature, where typically 10–10,000 times lower *α*S concentrations are used^18–24^. Interestingly, in a small number of previous studies using intermediate concentrations, a relatively rare subspecies has been observed^20,22^ that closely resembles the structure we report here for elevated concentrations. A previous EPR/fluorescence study based on three fluorescent labels has already hinted at a structural transition at lipid interfaces^25^. Here, we provide evidence for an upright orientation at elevated *α*S concentrations. This binding motif allows aggregation-prone middle regions^10,28^ of adjacent *α*S to to come in contact, which could be a key driver of amyloid aggregation of *α*S, possibly linked to the observed increase of aggregation-related toxicity at elevated *α*S concentrations^31^.

## Results

### VSFG experiment

**Fig. 1:**
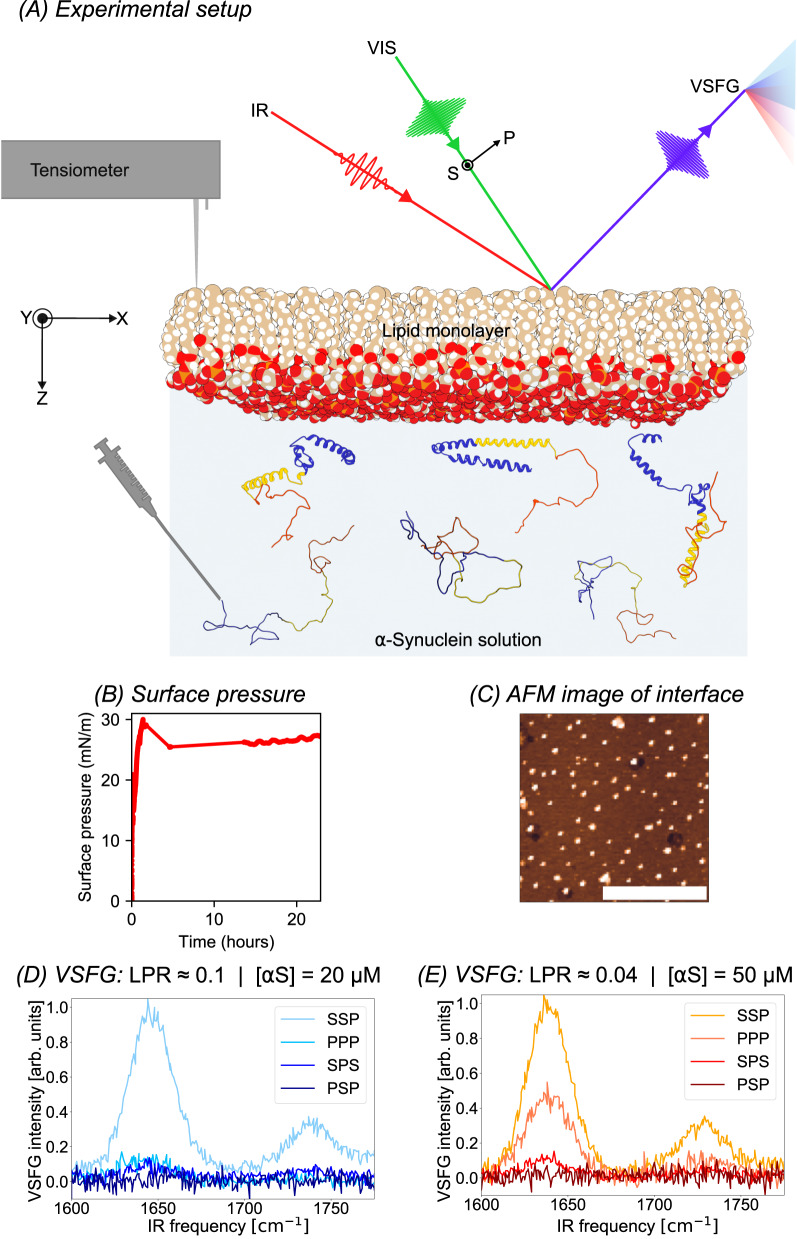
Overview of the experimental setup and main experimental results. **A** Schematic representation of the VSFG experiment. The protein solution is injected in the subphase under the lipid monolayer that is dropcast at the air–water interface. As was shown in previous studies^16,25^, *α*S converts from a randomly-coiled structure to a predominantly *α*-helical structure when it adsorbs to anionic lipid membranes. The protein adsorption is monitored by recording the surface pressure with a tensiometer, and with VSFG spectroscopy, which provides a frequency-dependent spectral signature of the interface. **B** Typical surface pressure curve for *α*S injection in the subphase below a DPPG monolayer. **C** AFM image of Langmuir deposition of the interface after 24 h of incubation that reveals the absence of fibrillar species. The spherical species that are observed (see also Supplementary Fig. 5) might be an artifact of the drying step in the AFM sample preparation, but they might also be oligomers formed in the *α*S aggregation process. The scale bar is 1 μm. **D**, **E** VSFG spectra at a relatively high (0.1) and low (0.04) lipid–protein ratio. The 0.1 LPR spectra are very similar to spectra recorded at an LPR of 37 (50 nM; see Supplementary Fig. 4). Source data are provided in the Source Data file.

We record orientation-sensitive VSFG spectra in the amide-I region (1600–1700 cm^−1^), as this mode is highly sensitive to the protein structure^39^. The signals are generated by overlapping an IR and a visible pulse in space and time and collecting the sum-frequency photons that are subsequently diffracted with a spectrograph on a CCD camera. By varying the polarizations of the sum-frequency, visible, and IR beams, one can record amide-I spectra in different VSFG polarization combinations, whose lineshapes and intensity ratios are strongly dependent on the structure and orientation (i.e., the conformation) of the protein^33^. The VSFG spectra are recorded with four different combinations of the polarizations of the two incoming (visible and IR) and one outcoming (SFG) beams. These polarization combinations can be divided into achiral and chiral combinations, where achiral polarization combinations can give SFG signals for both achiral and chiral structures, and the chiral combinations will only give a signal when the sample is chiral. We use a combination of several chiral and achiral polarization combinations to increase the information content of the experiment, which will later lead to a more unique fit with the structural model. Three of the recorded polarization combinations are achiral (SSP, i.e., S-polarized SFG, S-polarized visible, and P-polarized infrared, and PPP and SPS—see Fig. 1A) and is one chiral (PSP).

In Fig. 1A, the experimental setup is depicted. First, a DPPG lipid monolayer with a surface pressure of 15 mN/m is dropcast at the water surface (see Fig. 1B and Supplementary Fig. 2). Then *α*S is injected into the phosphate-buffered saline (PBS) subphase, leading to a final concentration of 50 nM, 20 μM, or 50 μM (LPR of ∼37, 0.091, or 0.037; see SI section 1.2 for the LPR calculations). At all these *α*S concentrations, surface binding, and assembly lead to an initial increase to a physiological surface pressure of approximately 30 mN/m^40^ within 5 h, after which it remains almost constant over ∼24 h (see Fig. 1B and Supplementary Fig. 2), indicating the formation of a stable layer. The strong binding is also visible in the recorded high signal-to-noise VSFG spectra of *α*S adsorbed to the DPPG monolayer (Fig. 1D, E and Supplementary Fig. 4), as weak and/or disordered binding will lead to very weak VSFG signals. The spectra show broad features centered around 1640 cm^−1^, which are typically associated with random-coil structures with a certain degree of order and/or *α*-helices^39^. The VSFG spectra are also relatively stable for the first ∼24 h, after which the protein and lipid signals start to decrease (see Supplementary Fig. 3A, B). While the intensity of the signal eventually drops over the ∼50 h incubation time, the normalized spectral lineshapes remain identical (Supplementary Fig. 3C). This signal evolution can be explained by an initial formation of an *α*S monolayer at the lipid interface, after which amyloidogenic protein-lipid aggregates are formed that detach from the interface, similar to what has previously been observed for *α*S, e.g., at an LPR of 10^28^, or (in a seeded fashion) at an LPR of 130^41^. AFM images taken of Langmuir depositions of the interface on mica after 24 h of incubation (Fig. 1C and Supplementary Fig. 5) confirm the absence of fibrillar aggregates. Because the Langmuir depositions are dried before we record the AFM images, we cannot rule out drying-induced oligomerization artifacts, but we observe 5- to 15-nm high spherical species that resemble previously observed isolated oligomers (e.g., those with a hydrodynamic radius of ∼12 nm^42^ and those with a TEM-derived diameter of ∼13 nm^43^), whose numbers appear to increase with incubation time. The absence of any changes in the normalized VSFG spectral lineshapes, while the AFM images show an oligomer-covered interface, could – if their presence is not a drying artifact – indicate that the oligomers are either centrosymmetric particles or that they have a random orientation with respect to the interface^33^.

Images of regions with defects recorded after 24 h of incubation (Supplementary Fig. 5) reveal the presence of areas where proteins have replaced lipids, which could (like the oligomerization) also be an artifact from the drying procedure, or a result of the high binding affinity of *α*S to highly curved lipid interfaces^30^ that are likely present at defects in the lipid membrane. Alternatively, the lipid replacement at long incubation times could indicate lipid co-aggregation, a scenario corroborated by the similar decrease in protein and lipid VSFG intensity as a function of time (see Supplementary Fig. 3 and Supplementary Fig. 5 for the associated AFM-based adhesion map that corroborates the AFM layer assignments) and by previous studies^15,44^. Interestingly, at *α*S concentrations identical to the concentration applied in the high-concentration experiment (∼50 μM) in our VSFG study, experiments with supported lipid bilayers deposited on an attenuated total reflection (ATR)–IR crystal under the protein solution did not show a decrease in the protein signal during the aggregation^45^. Instead, in this inverted (upside-down) geometry compared to our VSFG experiments, spectral lineshapes indicative of amyloids grow on a timescale of ∼10 h. This indicates that changes in the lipid composition (1:1 POPG:POPC vs. pure DPPG) or detachment of aggregates due to gravity might play a role in these experiments. In spite of differences in the ionic strength and exact lipid composition, the timescale of the aggregation in this ATR-IR experiment is comparable to the timescale of which the VSFG signal decreases, which seems to indicate that there is a correlation between *α*S aggregation and VSFG signal loss. In both the ATR-IR and our VSFG experiment, the lipid signal decreases during the incubation. Although some studies report that *α*S fibrils associate with anionic lipids such as DLPS and DMPS^44^, other studies using 1:1 POPC/POPG^41^ or DMPG^46^ lipids show very weak or negligible binding, respectively, of aggregated *α*S to lipid membranes compared to monomers. Therefore, it is likely that the aggregates formed by the protein–lipid co-aggregation have no preference to reside at either the remaining air-water or lipid-water interface, which is consistent with our observations.

The constant amide-I lineshapes of the normalized spectra (Supplementary Fig. 3C), and the AFM images that show the absence of fibrils (probably due to detachment) and that possibly indicate VSFG-invisibility of the oligomers (assuming the oligomers observed in the AFM images are not a drying artifact), make the applied experimental geometry well suited to study the binding mode of monomeric *α*S to DPPG lipids, undisturbed by aggregating species.

### Structural analysis by frame selection

#### Generation of structural hypotheses with MD simulations

To determine the molecular conformations of *α*S from the VSFG experiments, we have developed a new methodology in which spectral calculations are used to select frames from MD simulation trajectories. The MD simulations are used as a means to generate a large number of hypothetical conformations for which we calculate VSFG spectra that we compare with the experimental spectra. In order to generate the hypothetical structures, we run MD simulations of one *α*S molecule in water interacting with a DPPG monolayer, starting from a diverse set of eight starting conformations (see Supplementary Fig. 6) so that we sample a large portion of the *α*S conformational landscape near a DPPG interface. The starting structures include both kinked *α*-helical conformations based on the NMR-derived PDB entry 2KKW (SLAS-micelle bound *α*S), upright standing structures, and control simulations with disordered structures and structures penetrating the monolayer. The so-called horseshoe motif (PDB: 2KKW) is experimentally observed when *α*S is bound to sodium lauroyl sarcosinate (SLAS) micelles^21^, and refers to the horseshoe-shaped conformation composed of a kinked *α*-helix that spans from residue 1 to 91 that is tightly surface-bound, while its randomly coiled C-terminus is unbound. The fact that a similar motif is observed when *α*S interacts with SDS micelles^18^ (PDB: 1XQ8 (Human micelle-bound *α*S)), which indicates that it represents a typical *α*S folding near lipid-like interfaces, further validates including it in the sampling.

We run 16 MD production simulations (two repeats of 150 ns for each of the 8 starting structures, corresponding to a total simulation time of 2.4 μs) where we employ the DES-amber force field, recently developed for both folded and intrinsically disordered proteins (IDPs)^47^, together with the Slipids force field^48–50^. During the simulations, the *α*S starting structures partly unfold and reorient, thus generating a large dataset of 48.016 hypothetical *α*S conformations near a DPPG monolayer. We subsequently use excitonic spectral calculations^51,52^ to compare all the generated MD conformations with the experimentally observed VSFG spectra (SSP, SPS, PPP, and PSP) recorded during the first stable ∼15 h of incubation.

**Fig. 2:**
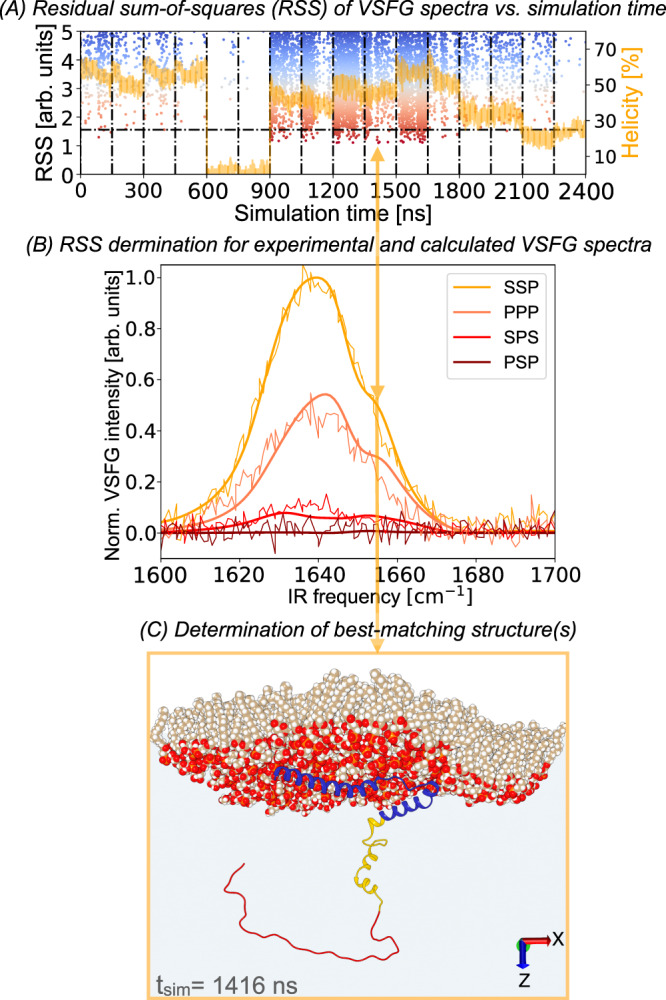
Example of frame-selection with the *ViscaSelect* algorithm (see “Code availability” section) on the MD simulation trajectories of *α*S near a DPPG lipid interface, using VSFG spectroscopy and spectral calculations. **A** An out-of-equilibrium (as can be seen by *α*S helicity traces in orange) MD simulation is performed 16 times, and each of the frames is compared with the experiments using spectral calculations. In this example, the residual-sum-of-squares (RSS) of the MD simulation is determined in comparison to the low-LPR experimental data and color-coded such that blue indicates a bad spectral match and red is a close spectral match. **B** Spectra from which the RSS-values are determined for the best-matching frame by taking the difference between the experimental (rugged, thin) spectra and calculated (smooth, thick) spectra at each frequency point, squaring these residuals and summing them. **C** The selected *α*S- and DPPG-structure of the best-matching conformation: the frame at a simulation time of 66 ns in the 10th trajectory of 150 ns, with the N-terminal region (residues 1–60) in blue, the NAC region (61–95) in yellow, and the C-terminal region (96–140) in red. Orange arrows connect the best-matching MD frame to associated the best-matching spectra and to the associated structure. Source data are provided in the Source Data file.

In previous studies where excitonic Hamiltonian VSFG calculations have been combined with MD simulations, snapshots of the lowest-energy states^36^, most-densely populated states^35^, or a representative set of MD snapshots^37^ have been used as the basis for the calculations. Other strategies have used full MD trajectories^34^ or particular trajectories out of a set of replicates^38^. The unfolded nature of medium-sized IDPs like *α*S requires relatively large MD systems that, in turn, make it prohibitively slow to obtain an equilibrium distribution of conformational states. While recent advances^47,53,54^ in MD force fields for IDPs have made MD simulations of both folded and disordered states accurate, sampling full MD folding trajectories requires very long simulations or accelerated sampling and is often only computationally possible for small peptides. Therefore, here we have expanded the methodology of combining VSFG with MD by using out-of-equilibrium MD simulations to generate a large set of protein conformations, after which we use spectral calculations (based on an amide-I one-exciton Hamiltonian model^33,37,38,52^; see “Methods” section for details) to determine which conformations give a match with the experimentally obtained spectra^55,56^. This removes the requirement of the MD simulation to fully converge as the spectral calculations can be used to select the MD-derived conformations that correctly describe the experiment via spectral comparison. The VSFG-MD frame-selection method (using the *ViscaSelect* algorithm) is illustrated in Fig. 2, which shows how the structures obtained from the MD trajectories are validated with spectral calculations, using their spectral similarity to the experimental VSFG spectra as the selection criterion.

#### Evaluating and selecting MD frames by VSFG spectral match

**Fig. 3:**
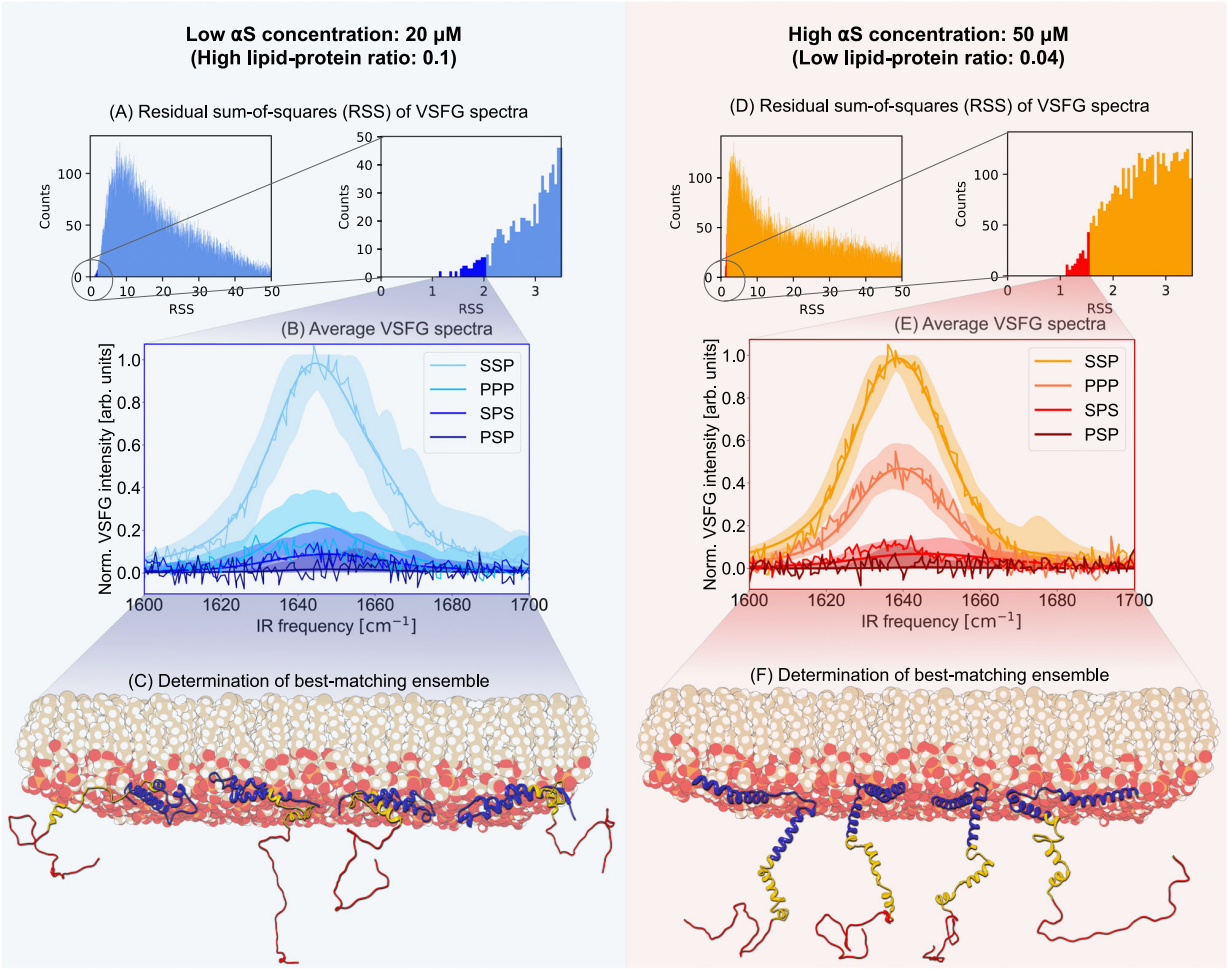
Application of the frame-selection methodology to the high-LPR (A–C) and low-LPR (D–F) datasets. **A**, **D** Histograms of the RSS values of all the MD-frames up to RSS = 50 (left), with a zoom-in on the best-matching ensemble (right; in dark-blue/dark-red) for which the calculated standard deviation is equal to the experimental standard deviations of the datasets composed from taking the averages of 10–50 spectra recorded with 5-min acquisition times each. This leads to RSS thresholds of 2.045 for the high-LPR experiment and 1.559 for the low-LPR experiment. **B**, **E** Experimental (rugged, thin lines) and calculated spectra for the best-matching frame (filled areas indicate the intensity range of all spectra within the best-matching ensemble, and the smooth, thick lines indicate the average spectrum of the best-matching ensemble). **C**, **F** Selections of typical structures found in the best-matching ensembles of the high- and low-LPR experiments, again with the N-terminal region (residues 1–60) in blue, the NAC region (61–95) in yellow, and the C-terminal region (96–140) in red. Structural inspection of the ensembles derived from this approach reveals a transition from flat-lying *α*S at low *α*S concentrations to an upright orientation at high-*α*S concentrations. Source data are provided in the Source Data file.

The evaluation of the experimental fitness of the MD conformations through the RSS allows us to select an ensemble of MD frames with the best spectral match. We set the cut-off of the ensembles such that the standard deviation of the spectra within the best-matching ensemble equals the experimental standard deviation. Thus we obtain two ensembles of MD frames, one for each experimental VSFG dataset. In Fig. 3, the spectral and structural ensembles on both sides of the transition are described: on the right for the low-LPR (high *α*S concentration) case, and on the left for the high-LPR (low *α*S concentration) case; here only depicted for the 20 μM dataset — the analysis for the 50 nM dataset gives a nearly identical result. On the top row of Fig. 3, the RSS values are given for the two cases; on the middle row, the range and average spectra of the ensembles are depicted, and on the bottom row, associated structures of each of the ensembles are shown. We note that the MD simulations do not explicitly incorporate the different LPR values. The same set of *α*S conformations is thus used as the collection to select from for both LPRs. In each case, an ensemble of best-matching structures is selected from this collection of frames based on the spectral match between the experimental and calculated VSFG spectra.

From the top row of Fig. 3, one can see that only a small fraction of the frames match the posed RSS criterion. The low- and high LPR ensembles contain 55 and 164 structures, respectively. Interestingly, there are no shared frames between the ensembles.

In the second row of Fig. 3, the transparent bands indicate the range of the spectra within the ensembles, which all show a reasonable overlap with the experimental spectra. Interestingly, their average spectra (solid lines) reproduce the experimental spectra very closely. This indicates that — as expected — the experimental spectrum is probably the result of an ensemble with a certain degree of variation in the tertiary structure and hydrogen bonding that is captured by the selected ensembles, which, upon averaging, leads to a close match with the observed spectra. The match of the weaker polarization combinations is slightly less good than the match with the SSP spectra, which can be understood from the fact that the selection — based on the total RSS of all polarization combinations — results in a higher sensitivity to stronger polarization combinations. The lack of any PSP intensity, which would originate from chiral amide-I normal modes, is reproduced well. The transparent band, which indicates the bandwidth, shows that some structures within the ensemble have a small non-zero chiral (PSP) response nonetheless, so including the PSP intensity in the frame selection criteria appears to help with filtering out frames with potentially larger chiral responses.

In the last row of Fig. 3, some typical structures are depicted (see Supplementary Fig. 8 for the 10 best matching structures) that are found within the ensembles. Visual inspection of the top-scoring conformations reveals a clear transition from a flat-lying *α*S to an upright orientation when going from a low *α*S concentration (high LPR) to a high *α*S concentration (low LPR). The absence of a chiral spectral response is consistent with these structures, as they are solely composed of random coils and *α*-helices, which are both known to be chiral-VSFG silent secondary structures in the amide-I region^57^.

### The interfacial structure of *α*S at low and high concentrations

**Fig. 4:**
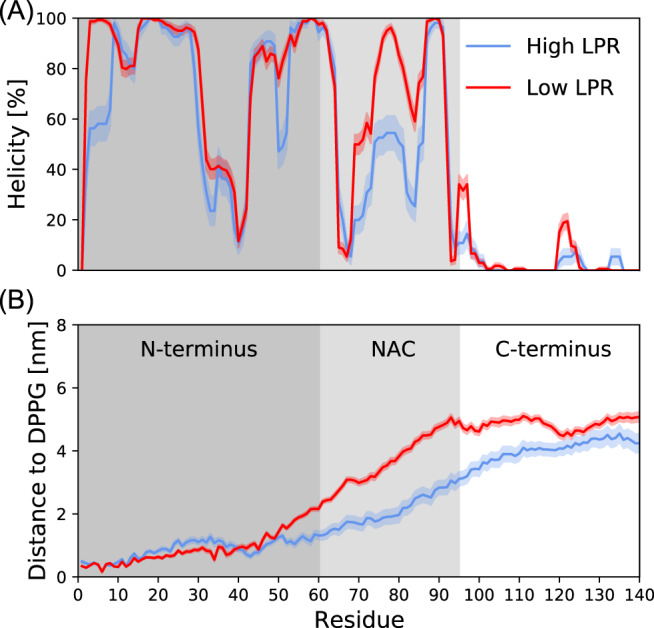
Comparison of structural features of the high- and low-LPR ensembles as a function of residue index. **A** The fraction of the ensemble that adopts a helical structure at the given residue, as determined with the simplified DSSP scheme, MDTraj v1.9.4^84^. **B** The average minimum distance from each residue to the nearest DPPG atom. For both **A** and **B**, the standard error of the mean within the ensembles is indicated by the transparent bands. Source data are provided in the Source Data file.

Now that the best-matching frames have been selected from the MD trajectories, one can obtain a quantitative structural image of the high- and low-LPR ensembles and determine to which extent there are commonalities and differences between the two ensembles. In Fig. 4, both the helicity (given as the fraction of the residues along the protein sequence that are adopting a helical structure within the ensembles) and the distance to the closest DPPG molecule are given for each protein residue. The plots reveal that both the low- and high-LPR ensembles contain several broken helices in the residue 1–95 segment, while the low-LPR ensemble contains conformations that are, on average, further away from the DPPG surface compared to the high-LPR ensemble conformations — in particular in the residue 50–120 segment. The small standard error of the mean, especially on the distance to the nearest DPPG molecule, indicates that the frame-selection method yields ensembles that are characterized by a structural similarity (see Supplementary Fig. 9 for the distances of each of the structures in the ensembles). This allows one to obtain a good understanding of what structural features remain the same and which change when the protein concentration is varied.

While the proteins within the high- and low-LPR ensembles adopt a very different orientation, surprisingly, the regions that adopt a helical structure are similar in the two ensembles. This could indicate a cooperative folding behavior along the protein chain, where — even in the protruding helical structure adopted at high protein concentrations — N-terminal lipid binding and subsequent local helix formation results in secondary structure changes further down the chain. This is supported in the *α*S sequence (Uniprot: P37840) by several imperfect KTKEGV repeats in the N-terminus that promote helix formation.

Summarizing, Fig. 4 supports what was observed in the visual inspection of the best-matching structures for the two LPRs (Fig. 3 and Supplementary Fig. 8): (1) for all proteins in each of the two ensembles, the best-matching structures all have an intermediate degree of helicity (see also Supplementary Fig. 10 for the secondary structure per protein segment), divided over several broken helices, and a tight lipid binding by the first ∼50 N-terminal residues, and (2) a clear distinction between the two ensembles is the orientation of the remainder of the protein (especially of the NAC region), where an upright orientation is an important prerequisite for a low-RSS fit to the low-LPR measurements, while a flat orientation is a prerequisite for a good spectral match with the high-LPR measurements.

## Discussion

Here, we will first compare our derived structural ensembles with previously published models. The derived high-LPR (low *α*S concentration) ensemble shows remarkable similarities to reported literature structures obtained at similar LPRs, while the low-LPR (high *α*S concentration) ensemble, derived for a concentration that is less accessible with most experimental techniques, deviates significantly.

The helicity in both the high- and low-LPR ensemble is comparable to the starting, horseshoe-shaped conformation, which has been observed with NMR for *α*S adsorbed to sodium dodecanol sulfate (SDS)^18^ and NMR/EPR for *α*S adsorbed to SLAS^21^ experiments at low protein concentrations. However, the close lipid association observed in the latter study, performed at a lipid-to-protein ratio of ∼30 and higher^21,22^, is only seen for the high-LPR (∼0.1 and ∼37) VSFG-derived ensembles. Other EPR and NMR studies at lower relative *α*S concentrations also showed tightly lipid-bound, largely *α*-helical structures near phospholipid vesicles, rod-like SDS micelles, or lipid bicelles with LPRs in the range of 50–2000^19,20,23^, albeit in these studies a straight *α*-helix was observed, similar to the structure observed with NR at LPRs ranging between 100 and 300^24^. Interestingly, two NMR studies that also investigated relatively high *α*S concentrations (LPRs of around 1^20^ and 30^22^, respectively), closer to the here-employed low LPR value of ∼0.04, found a small second *α*S population that is less lipid-associated in co-existence with the closely lipid-associated species. This less lipid-associated *α*S conformation is likely the same binding motif we derive from our low-LPR experiments.

Obviously, the exact *α*S concentration where the protein conformation undergoes the transition from flat to upright will depend on many factors, like surface curvature, temperature, lipid composition, lipid phase, and salt concentration. This will lead to different transition concentrations, for example, for experimental systems utilizing SUVs (e.g., studies refs. ^20,22^) vs. flat Langmuir monolayers (this study), for studies using different lipid mixtures (although interestingly, there is no difference in the low *α*S-concentration binding mode to pure POPG vesicles and to vesicles composed of a 5:3:2 of DOPE:DOPS:DOPC lipid mixture^22^), and when applying lower temperatures (e.g., −19 or +4 °C^22^ or 10–20 °C^20^) vs. the 23 °C applied in the current study. It is nevertheless insightful to compare the results of various studies, e.g., the various reports on the high-LPR (low concentration) structure of *α*S show great similarities while providing new information from the various techniques. It is, thus, for example, interesting to note that the authors of ref. ^22^ observe that even at relatively low *α*S concentrations (i.e., high LPRs), the contacts between the NAC region and the lipids have a transient nature, while also in the high-LPR ensemble, we see a few structures that indeed show an upright NAC orientation (see Supplementary Fig. 8), but other than that, the frame-selection method in its here-presented form will not provide information on the dynamics of the conformational states. The previous and current studies are thus complementary to each other, and our results bring new and potentially important findings to the table, especially in providing new structural detail of the concentration-dependent structural transition^25^, which might bear relevance through a potentially disease-related elevation of the *α*S concentration^31^ that can thus lead to membrane-adsorbed *α*S structures in which the aggregation-prone NAC regions are in close proximity.

**Fig. 5:**
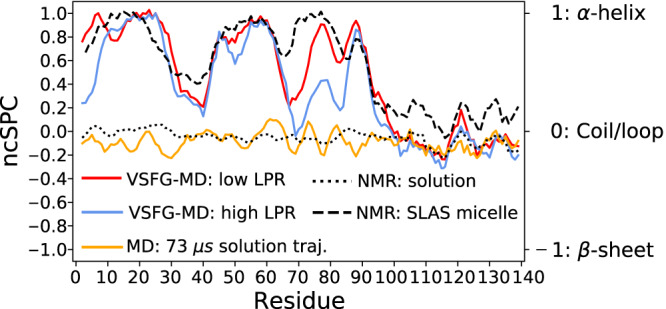
Secondary structure propensities calculated from NMR chemical shifts. Cumulative scores from the ncSPC webserver^59^ of the best-matching MD-VSFG ensembles for the low- (red) and high- (blue) LPR ensembles are depicted from predicted chemical shifts (SPARTA+^58^) and of an MD simulation of disordered *α*S in solution^53^ (yellow). Experimental chemical shifts of *α*S bound to SLAS micelles (black dashed line, BMRB: 16302) and in solution (black dotted line, BMRB: 25227) are shown for reference. Source data are provided in the Source Data file.

To structurally compare our results directly with the previously-proposed models, we calculate NMR chemical shifts (CSs) and VSFG spectra based on previously-reported structures. For this, we analyze the frames from the present simulation within the best-matching ensembles, defined such that their standard deviation is similar to the experimental one (see previous sections). NMR CSs are then calculated from each MD frame using the SPARTA+^58^ artificial neural network, and the average CS of each MD-VSFG ensemble was uploaded to the *neighbor-corrected structure propensity calculator*^59^ (ncSPC) webserver^60^ to obtain a secondary structure propensity score (Fig. 5). An ncSPC score of 1 indicates a fully formed helix, -1 indicates *β*-structure, and 0 is a coil/loop disordered structure. We do the same for the CSs obtained in the SLAS micelle-associated *α*S NMR experiment (BRMB 16302)^21^, which we also used as one of the starting structures (see Supplementary Fig. 6), and, as references, for disordered *α*S in solution as determined with NMR (BRMB 25227)^61^ and with a 73 μs solution MD trajectory kindly provided by Robustelli et al.^53^. The ncSPC propensity scores yield a very similar picture for the high- and low-LPR ensembles as the DSSP helicity values depicted in Fig. 4A, which demonstrates the consistency between the two methods to quantify the secondary structure. The experimental data for both low- and high LPR experiments is again most consistent with a sequence of broken *α*-helices, which follows the secondary-structure trend of *α*S adsorbed to SLAS micelles surprisingly well. Such a ∼50/50 *α*-helical/random-coil conformation has also been observed with other techniques, like UV-CD at a high LPR of 750^25^ and, at a similarly low LPR value (∼0.033; see SI for LPR determination) as the one we employ in the low-LPR experiment^45^, with ATR-IR.

Because the VSFG signal is not only sensitive to secondary structure but also to tertiary structure and protein orientation, we further investigate these aspects in relation to literature models by calculating the VSFG spectra of the straight^19,20,24,62^ and kinked^18,21^, mainly *α*-helical structures and comparing them with the experimental high- and low-LPR datasets for all possible protein orientations (see Supplementary Fig. 11). Interestingly, for the high-LPR VSFG spectra, we find a close spectral match for the flat-lying orientations that have been observed before for the two protein structures, which corroborates the results obtained with the frame-selection method (Figs. 3 and 4). In contrast, for the low-LPR spectra, the RSS values are generally significantly larger for both the straight and kinked helices, especially for the protein orientations reported in the literature, which have RSS values at least 10 times as large as the lowest RSS values that we find for the MD frames. The large RSS values obtained for these literature structures also substantiate the result from the frame-selection method at the low LPR of 0.04, as the derived N-terminally bound and otherwise protruding helical structure is inconsistent with the two structural models that are available in the literature, which have been obtained for higher (∼1^20^–∼2000^19^) LPR values^18–21,24,62^.

Neuronal cell membranes contain significant fractions of neutral lipids like cholesterol, a majority of zwitterionic lipids like PC and PE, and a minority of anionic lipids like PI, PA, and PS^63,64^. Reducing anionic membrane charge from the 100% anionic lipid contents used in this study is known to lead to decreased interaction with *α*S as has been shown with, e.g., UV-CD spectroscopy^65^, fluorescence correlation spectroscopy^30^, and voltage-dependent anion channel nanopore measurements^66^, indicating that *α*S-lipid membrane interaction is driven by negatively charged lipid species. But as shown by Fusco et al.^22^, the low *α*S concentration binding mode of the rigid parts of the protein is actually similar to vesicles composed of pure POPG and to vesicles composed of a 5:3:2 of DOPE:DOPS:DOPC lipid mixture, which shows that the anionic vs. mixed lipid composition does not strongly affect the *α*S conformation. The conformation of *α*S bound to zwitterionic lipids will probably mainly differing from the orientation derived in the present study by the aspect that the negatively-charged C-terminus will not be pointing away from the lipid interface so much^66^ due to charge repulsion that occurs with negatively charged lipids like DPPG.

Now, we will focus on the implications for health and disease. The strong experimental VSFG signals indicate that at low- (∼50 nM), approximately physiological (∼20 μM^29^), and elevated (∼50 μM) protein concentrations, *α*S forms densely packed monolayers at lipid interfaces. Recently, *α*S has been shown to adsorb to lipids in a strongly cooperative manner^67^, which enhances the formation of such high-density *α*S layers. As previously suggested^22^, the upright *α*S conformation — here derived at elevated *α*S concentrations — may be relevant both in a healthy context where it can form a bridge between lipid vesicles or between vesicles and membranes in order to maintain the stasis of neuronal vesicles. It has also been shown that such a protruding form of *α*S plays a role in the clustering of synaptic vesicles^68^. In the elevated-concentration conformation, the C-terminus of *α*S is maximally available for synaptobrevin-2 binding^69,70^, thereby promoting SNARE-complex assembly, which facilitates membrane fusion. The derived high-concentration structure can also be consistent with the relatively-high concentration EPR observations that reveal that the hydrophobic side of the αS molecules is shielded from the solvent^62^, only not interpreted such that the αS molecules lie flat on the lipid membrane, but instead form “bundles” at the interface^20^. In such a conformation, the hydrophobic sides of the amphipatic helices could come together, potentially interacting with extracted lipid tails. Furthermore, as also previously suggested^20,71^, it could be a particularly relevant structure in the development of synucleopathies as well, given the fact that this upright structure results in a high local concentration where the amyloidogenic NAC regions of the lipid-bound *α*S molecules line up, which can explain the catalytic effect that lipids have on *α*S aggregation^16^. We speculate that the fact that the eventually formed fibril structure is not sensitive to the LPR^72^, combined with the fact that the solely N-terminally bound *α*S structures reported in this study have been observed in co-existence with the flat-lying *α*S structure^20,22^, could indicate that the extended structure purely observed here at elevated *α*S concentrations, is the intermediate structure that leads to lipid-catalyzed fibril formation at all LPRs.

Finally, we provide some summarizing remarks. To study the interaction of *α*S with lipid interfaces as a function of concentration, we have developed an effective approach for extracting structural information from experimental VSFG spectra using a combination of non-equilibrium MD simulations and spectral calculations. We apply it to investigate *α*S binding to lipid surfaces, from low concentrations — which have been studied previously using other methods — to physiological and elevated protein concentrations. The method has allowed us to study the structure and orientation of *α*S with a high structural resolution.

**Fig. 6:**
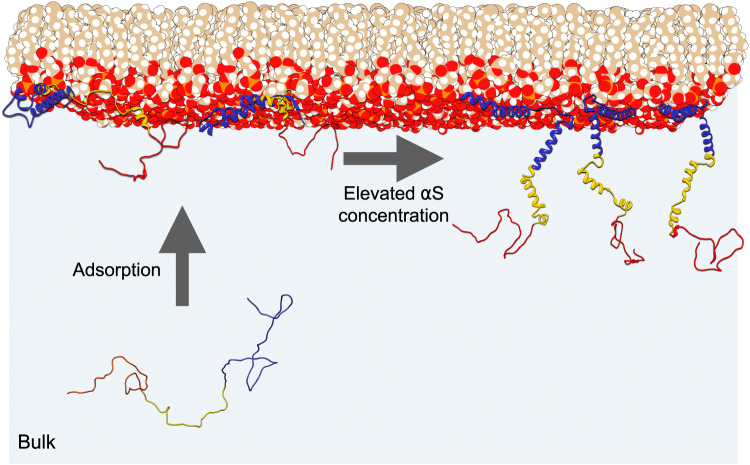
Effect of protein concentration on *α*S conformation at lipid surfaces. At relatively low concentrations, *α*S converts from a randomly-coiled structure to a flat-lying, mainly *α*-helical structure after adsorption to the lipid membrane from the bulk^21^, but at elevated concentrations, the protein adopts an upright orientation in which the aggregation-prone NAC regions come in close proximity of each other.

Using the *ViscaSelect* algorithm to implement the frame-selection method, we reveal a structural transition as a function of concentration from a low *α*S conformation in which it adopts a flat binding pose with its helices parallel to the lipid surface to a more upright orientation of the proteins as the *α*S concentration increases (see Fig. 6). The low-concentration (high-LPR) structure is consistent with the derived structures at similarly high LPRs, which benchmarks the here-presented method. The degree of helicity observed in the best-matching ensemble is also comparable to what has previously been observed.

In the upright conformation, derived here for elevated *α*S concentrations, the first ∼50 N-terminal residues anchor the protein to the lipid membrane, similar to what we derive for the relatively lower-*α*S-concentration conformations. However, the upright orientation of the remainder of the protein (especially of the aggregation-probe middle *NAC* region) provides an opportunity for *α*S to to engage in more extensive intermolecular contacts and may pave the way for subsequent aggregation. This molecular mechanism can explain both the concentration-dependent aggregation catalysis by lipids^16^ and the aggregation-related toxicity observed at elevated *α*S concentrations^31,73^.

## Methods

### *α*S expression and purification

Purified *α*S was expressed and purified as follows, and as also described in^74^. The plasmid vector pET11-d was used to express *α*S in *Escherichia coli* BL21 (DE3). The cells were then pelleted by 20 min of centrifugation at 2657 × *g*, at 4 °C. The pellet of 1 L of culture was resuspended in 100 mL of osmotic shock buffer (composed of 30 mM Tris-HCl, 40% sucrose, and 2 mM EDTA at pH 7.2) and incubated for 10 min. Subsequently, the suspension was centrifuged for 30 min at 9000 × *g* and 20 °C. The resulting pellet was then resuspended in 90 mL of ice-cold deionized water, and 40 μL of saturated MgCl_2_ was added. Subsequently, the pellet was incubated on ice for 3 min, and the supernatant, containing the periplasmic preparation, was collected by 20 min centrifugation at 9000 × *g* and 4 °C. The periplasmic preparation was subjected to acid precipitation with drop-wise addition of 1 M HCl to a final pH level of 3.5 and then incubated for 5 min. The supernatant was collected by 20 min of centrifugation at 9000 × *g* and 4 °C. The pH of the supernatant was immediately adjusted to pH 7.5 with the drop-wise addition of 1 M NaOH. The solution was filtered (0.45 μm) and loaded on a Q-Sepharose column (HiTrap Q HP) pre-equilibrated with 20 mM Tris-HCl pH 7.5. The column was washed with three column volumes of 0.1 M NaCl in buffer followed by elution of *α*S with a NaCl gradient from 0.1 to 0.5 M. SDS-PAGE analysis was used to identify fractions with *α*S and to ensure protein purity. Finally, the *α*S was dialyzed exhaustively against deionized water, lyophilized, and stored at −20 °C.

Prior to use, the lyophilized powder was dissolved in PBS (0.01 M phosphate buffer, 0.0027 M KCl, and 0.137 M NaCl, pD 7.2, Sigma Aldrich) in D_2_O (99.9%D, Eurisotop) to avoid interference from H_2_O bending modes. The *α*S solution was prepared either by filtration with 100 kDa Nanosep filters (Pall Corporation, USA) or without this filtration step because we observed that this did not affect any of the kinetic or spectral features of the measurements (see Supplementary Fig. 13).

### Lipid monolayer preparation and protein injection

A lipid monolayer of DPPG (1,2-dipalmitoyl-sn-glycero-3-phospho-(1'-rac-glycerol) (sodium salt), Avanti Polar Lipids) is assembled at the air–water interface within a 2 mL trough at room temperature. The DPPG is first dissolved in chloroform and then dropcast at the air–water interface to an initial surface pressure of ∼15 mN/m (see Supplementary Fig. 2), corresponding to a liquid-condensed (LC) phase^75^. After equilibrium, VSFG spectra are recorded on the pure lipid monolayer (see Supplementary Fig. 3). After injection of the protein solution, the surface pressure increased to ∼30 mN/m (see Supplementary Fig. 2).

The motivation for performing these measurements with a DPPG monolayer at ∼30 mN/m are as follows: (1) we have to use DPPx lipids instead of unsaturated lipids (like DOPx and POPx, which are more abundant in biological membranes^76^) because the C=C modes are close to the amide-I band^77^ that we want to use to determine the protein conformation, and mainly because these kinds of lipids are very unstable and can oxidize quickly when exposed to air. Because of the long measurement times required to follow the *α*S aggregation (tens of hours), the oxidative changes in the model surface would render the experiments less reliable and reproducible. (2) We aim for an area per lipid that is comparable to the value in an average idealized mammalian plasma membrane (50 Å^2^/molecule) and obtained this by measuring at the surface pressure to 30 mN/m^78^, which is also considered to be the physiological surface pressure^40^. (3) Many other biomimetic monolayer studies have also been performed around this surface pressure (e.g., refs. ^40,79,80^), so for comparability with respect to such studies, the choice for 30 mN/m is ideal. (4) Anionic DPPG lipids bind relatively strongly to *α*S^30^, and in aging mice brains, the levels of various anionic lipids are higher than in young mice^64^, so there might be biomedical relevance of such a relatively negative lipid interface as well. (5) Finally, the binding mode of *α*S — at least at relatively low *α*S concentrations — appears to be similar to vesicles composed of pure POPG as it is to vesicles composed of a 5:3:2 of DOPE:DOPS:DOPC lipid mixture^22^, which indicates that the anionic component of the lipid mixture dominates the protein–lipid interaction.

In the 50 nM, 20 μM, and 50 μM experiments, the protein was dissolved at 250 nM, 100, and 250 μM concentration, respectively, in 400 μL of PBS-D_2_O buffer before injection into a total subphase of 2 mL below the lipid monolayer, while stirring the subphase with a magnetic stirring bar. The concentration before protein injection of each experiment was measured by UV–vis spectroscopy using the absorbance at 280 nm (NanoDrop 2000c, Thermo Scientific).

### VSFG spectroscopy

The SFG setup has been previously described in ref. ^81^. In short, a narrowband (FWHM ∼15 cm^−1^) visible beam was temporally and spatially overlapped with a broadband IR beam at the sample surface, and the SFG signal was focused into a spectrograph and detected by an EMCCD camera. VSFG spectra were recorded between 1600 and 1800 cm^−1^ in SSP (S-SF, S-visible, P-IR), PPP, SPS, and PSP polarization combinations. All spectra were background subtracted and normalized using a reference spectrum obtained from gold. The various polarization combinations were recorded in a sequential manner, frequently going back to the SSP polarization combination in order to monitor the development of the overall signal intensity (see Supplementary Fig. 3).

### MD simulations

Eight systems of *α*S in different conformations and in contact with a DPPG monolayer were created (see Supplementary Fig. 6) using the CHARMM-GUI webserver^82^. MD simulations were performed using GROMACS^83^ v2021.4. We use the DES-amber force field^47^ for protein, water, and ions and the Slipid forcefield^48–50^ for lipids. Slipids are compatible with the AMBER99SB-ILDN force field branch that DES-amber is built upon. After minimization and 10 ns equilibration of the DPPG monolayer, production runs of 150 ns were simulated for each of the eight systems, with two repeat simulations in each. Frames were saved every 50 ps, totaling 48.016 individual *α*S conformations. See Supplementary Information for more details.

### Spectral calculations

The spectral calculations are based on an excitonic Hamiltonian approach developed and first described in ref. ^52^ and in detail in the Supplementary Information. We performed the calculations for a total of 48.016 different frames, with varying protein structures and orientations in each frame. Because we did not find an improvement in the spectral match when we averaged over multiple frames (see Supplementary Table 2), all presented spectral calculations are performed by directly obtaining the eigenmodes from each frame by evaluating the excitonic Hamiltonian model. The residual sum-of-squares (RSS) difference between the experimental and calculated spectra was used to find the frames that describe the experimental response best.

### Statistics and reproducibility

All VSFG, AFM, and surface-pressure measurements have been performed twice. The derived structural ensembles are robust over trajectories starting from different starting structures (given the similarity of, e.g., the 10 best-matching structures that come from various trajectories with different starting structures, with generally similar helicity-RSS trends depicted in Supplementary Fig. 12), the assumed Lorentzian normal mode width (up to a full-width-at-half-max of 12 cm^−1^, see Supplementary Table 3), and over reproduced experimental datasets (Supplementary Fig. 13), in that the qualitative conclusion remains the same irrespective of the parameters. We also explored whether averaging multiple frames would result in a better match by using an average over 10 and 100 successive frames (see Supplementary Table 4), but as the spectral match from single structures are already very good, it turned out to be impossible to achieve lower RSS values, so for the sake of simplicity we have focused on single structure results. Experimental modifications, like the removal of the DPPG monolayer from the air–water interface, result in markedly different best-matching structural ensembles (see Supplementary Fig. 14).

### Reporting summary

Further information on research design is available in the Nature Portfolio Reporting Summary linked to this article.

### Supplementary information

Supplementary Information

Reporting Summary

### Source data

Source Data

## Data Availability

The data generated in this study are provided in the Source Data file. The data used in this study are also available in the Zenodo database under accession code 7916728 [10.5281/zenodo.7916728]. Finally, the PDB structures can be found in the protein database, see in particular 2KKW (SLAS-micelle bound *α*S) and 1XQ8 (Human micelle-bound *α*S). Source data are provided in this paper.

## Code availability

The code used to calculate VSFG spectra and the *ViscaSelect* algorithm used to perform the frame-selection method is available from the authors upon reasonable request and on the Visca GitHub. We are currently working on making all code available in a GUI-based user-friendly fashion, which will be available for download from the same GitHub.

## References

[1] Chiti F, Dobson CM (2006). Protein misfolding, functional amyloid, and human disease. Annu. Rev. Biochem..

[2] Eisenberg D, Jucker M (2012). The amyloid state of proteins in human diseases. Cell.

[3] Knowles TP, Vendruscolo M, Dobson CM (2014). The amyloid state and its association with protein misfolding diseases. Nat. Rev. Mol. Cell Biol..

[4] Breydo L, Wu JW, Uversky VN (2012). *α*-Synuclein misfolding and Parkinson’s disease. Biochim. Biophys. Acta.

[5] Wong YC, Krainc D (2017). *α*-synuclein toxicity in neurodegeneration: mechanism and therapeutic strategies. Nat. Med..

[6] Singh SK, Dutta A, Modi G (2017). *α*-Synuclein aggregation modulation: an emerging approach for the treatment of Parkinson’s disease. Fut. Med. Chem..

[7] Alafuzoff I, Hartikainen P (2018). Alpha-synucleinopathies. Handb. Clin. Neurol..

[8] Miraglia F, Ricci A, Rota L, Colla E (2018). Subcellular localization of alpha-synuclein aggregates and their interaction with membranes. Neural Regen. Res..

[9] Ruipérez V, Darios F, Davletov B (2010). Alpha-synuclein, lipids and Parkinson’s disease. Prog. Lipid Res..

[10] Iyer A, Claessens MM (2019). Disruptive membrane interactions of alpha-synuclein aggregates. Biochim. Biophys. Acta.

[11] den Hartog Jager WA (1969). Sphingomyelin in Lewy inclusion bodies in Parkinson’s disease. Arch. Neurol..

[12] Gai W (2000). In situ and in vitro study of colocalization and segregation of *α*-synuclein, ubiquitin, and lipids in Lewy bodies. Exp. Neurol..

[13] Shahmoradian SH (2019). Lewy pathology in Parkinson’s disease consists of crowded organelles and lipid membranes. Nat. Neurosci..

[14] Grey M, Linse S, Nilsson H, Brundin P, Sparr E (2011). Membrane interaction of *α*-synuclein in different aggregation states. J. Parkinson’s Dis..

[15] Hellstrand E, Nowacka A, Topgaard D, Linse S, Sparr E (2013). Membrane lipid co-aggregation with *α*-synuclein fibrils. PloS ONE.

[16] Galvagnion C (2015). Lipid vesicles trigger *α*-synuclein aggregation by stimulating primary nucleation. Nat. Chem. Biol..

[17] Galvagnion C (2017). The role of lipids interacting with *α*-synuclein in the pathogenesis of Parkinson’s disease. J. Parkinson’s Dis..

[18] Ulmer TS, Bax A, Cole NB, Nussbaum RL (2005). Structure and dynamics of micelle-bound human *α*-synuclein. J. Biol. Chem..

[19] Georgieva ER, Ramlall TF, Borbat PP, Freed JH, Eliezer D (2008). Membrane-bound α-synuclein forms an extended helix: long-distance pulsed ESR measurements using vesicles, bicelles, and rodlike micelles. J. Am. Chem. Soc..

[20] Bodner CR, Dobson CM, Bax A (2009). Multiple tight phospholipid-binding modes of *α*-synuclein revealed by solution NMR spectroscopy. J. Mol. Biol..

[21] Rao JN, Jao CC, Hegde BG, Langen R, Ulmer TS (2010). A combinatorial NMR and EPR approach for evaluating the structural ensemble of partially folded proteins. J. Am. Chem. Soc..

[22] Fusco G (2014). Direct observation of the three regions in *α*-synuclein that determine its membrane-bound behaviour. Nat. Commun..

[23] Antonschmidt L (2021). Insights into the molecular mechanism of amyloid filament formation: Segmental folding of *α*-synuclein on lipid membranes. Sci. Adv..

[24] Hellstrand E (2013). Adsorption of *α*-synuclein to supported lipid bilayers: positioning and role of electrostatics. ACS Chem. Neurosci..

[25] Shvadchak VV, Yushchenko DA, Pievo R, Jovin TM (2011). The mode of *α*-synuclein binding to membranes depends on lipid composition and lipid to protein ratio. FEBS Lett..

[26] Galvagnion C (2016). Chemical properties of lipids strongly affect the kinetics of the membrane-induced aggregation of *α*-synuclein. Proc. Natl Acad. Sci..

[27] Hoover BM (2021). Membrane remodeling and stimulation of aggregation following *α*-synuclein adsorption to phosphotidylserine vesicles. J. Phys. Chem. B.

[28] Iyer A, Petersen NO, Claessens MM, Subramaniam V (2014). Amyloids of alpha-synuclein affect the structure and dynamics of supported lipid bilayers. Biophys. J..

[29] Wilhelm BG (2014). Composition of isolated synaptic boutons reveals the amounts of vesicle trafficking proteins. Science.

[30] Middleton ER, Rhoades E (2010). Effects of curvature and composition on *α*-synuclein binding to lipid vesicles. Biophys. J..

[31] Olivares D, Huang X, Branden L, Greig NH, Rogers JT (2009). Physiological and pathological role of alpha-synuclein in Parkinson’s disease through iron mediated oxidative stress; the role of a putative iron-responsive element. Int. J. Mol. Sci..

[32] Yan ECY, Wang Z, Fu L (2015). Proteins at interfaces probed by chiral vibrational sum frequency generation spectroscopy. J. Phys. Chem. B.

[33] Hosseinpour S (2020). Structure and dynamics of interfacial peptides and proteins from vibrational sum-frequency generation spectroscopy. Chem. Rev..

[34] Carr JK, Wang L, Roy S, Skinner JL (2015). Theoretical sum frequency generation spectroscopy of peptides. J. Phys. Chem. B.

[35] Bellucci L (2016). The interaction with gold suppresses fiber-like conformations of the amyloid *β* (16–22) peptide. Nanoscale.

[36] Harrison ET, Weidner T, Castner DG, Interlandi G (2017). Predicting the orientation of protein G B1 on hydrophobic surfaces using Monte Carlo simulations. Biointerphases.

[37] Lu H (2019). Peptide-controlled assembly of macroscopic calcium oxalate nanosheets. J. Phys. Chem. Lett..

[38] Alamdari S (2020). Orientation and conformation of proteins at the air–water interface determined from integrative molecular dynamics simulations and sum frequency generation spectroscopy. Langmuir.

[39] Barth A (2007). Infrared spectroscopy of proteins. Biochim. Biophys. Acta.

[40] Ameziane-Le Hir S (2014). Cholesterol favors the anchorage of human dystrophin repeats 16 to 21 in membrane at physiological surface pressure. Biochim. Biophys. Acta.

[41] Chaudhary H, Subramaniam V, Claessens MM (2017). Direct visualization of model membrane remodeling by α-synuclein fibrillization. ChemPhysChem.

[42] Lorenzen N, Lemminger L, Pedersen JN, Nielsen SB, Otzen DE (2014). The N-terminus of *α*-synuclein is essential for both monomeric and oligomeric interactions with membranes. FEBS Lett..

[43] Paslawski W (2014). High stability and cooperative unfolding of *α*-synuclein oligomers. Biochemistry.

[44] Galvagnion C (2019). Lipid dynamics and phase transition within *α*-synuclein amyloid fibrils. J. Phys. Chem. Lett..

[45] Fallah MA (2017). Simultaneous IR-spectroscopic observation of *α*-synuclein, lipids, and solvent reveals an alternative membrane-induced oligomerization pathway. ChemBioChem.

[46] Ramakrishnan M, Jensen PH, Marsh D (2006). Association of *α*-synuclein and mutants with lipid membranes: spin-label ESR and polarized IR. Biochemistry.

[47] Piana S, Robustelli P, Tan D, Chen S, Shaw DE (2020). Development of a force field for the simulation of single-chain proteins and protein–protein complexes. J. Chem. Theory Comput..

[48] Jambeck JP, Lyubartsev AP (2012). Derivation and systematic validation of a refined all-atom force field for phosphatidylcholine lipids. J. Phys. Chem. B.

[49] Jambeck JP, Lyubartsev AP (2013). Another piece of the membrane puzzle: extending slipids further. J. Chem. Theory Comput..

[50] Grote F, Lyubartsev AP (2020). Optimization of slipids force field parameters describing headgroups of phospholipids. J. Phys. Chem. B.

[51] Hamm, P. & Zanni, M. *Concepts and Methods of 2D Infrared Spectroscopy*. (Cambridge University Press, 2011).

[52] Roeters SJ (2013). Determining in situ protein conformation and orientation from the amide-I sum-frequency generation spectrum: theory and experiment. J. Phys. Chem. A.

[53] Robustelli P, Piana S, Shaw DE (2018). Developing a molecular dynamics force field for both folded and disordered protein states. Proc. Natl Acad. Sci. USA.

[54] Piana S, Donchev AG, Robustelli P, Shaw DE (2015). Water dispersion interactions strongly influence simulated structural properties of disordered protein states. J. Phys. Chem. B.

[55] Snow CD, Zagrovic B, Pande VS (2002). The Trp cage: folding kinetics and unfolded state topology via molecular dynamics simulations. J. Am. Chem. Soc..

[56] Duan L (2019). Accelerated molecular dynamics simulation for helical proteins folding in explicit water. Front. Chem..

[57] Fu L (2015). Characterization of parallel *β*-sheets at interfaces by chiral sum frequency generation spectroscopy. J. Phys. Chem. Lett..

[58] Shen Y, Bax A (2010). SPARTA+: a modest improvement in empirical NMR chemical shift prediction by means of an artificial neural network. J. Biomol. NMR.

[59] Tamiola K, Mulder FA (2012). Using NMR chemical shifts to calculate the propensity for structural order and disorder in proteins. Biochem. Soc. Trans..

[60] Neighbor-corrected structure propensity calculator (ncSPC) web-server, accessed February. https://st-protein02.chem.au.dk/ncSPC/ (2021).

[61] Porcari R (2015). The H50Q mutation induces a 10-fold decrease in the solubility of *α*-synuclein. J. Biol. Chem..

[62] Jao CC, Hegde BG, Chen J, Haworth IS, Langen R (2008). Structure of membrane-bound *α*-synuclein from site-directed spin labeling and computational refinement. Proc. Natl Acad. Sci. USA.

[63] Wenk LLM (2009). 9 Neuronal membrane lipids–their role in the synaptic vesicle cycle. Handb. Neurochem. Mol. Neurobiol..

[64] Rappley I (2009). Lipidomic profiling in mouse brain reveals differences between ages and genders, with smaller changes associated with *α*-synuclein genotype. J. Neurochem..

[65] Kjaer L, Giehm L, Heimburg T, Otzen D (2009). The influence of vesicle size and composition on *α*-synuclein structure and stability. Biophys. J..

[66] Jacobs D (2019). Probing membrane association of *α*-synuclein domains with VDAC nanopore reveals unexpected binding pattern. Sci. Rep..

[67] Makasewicz K (2021). Cooperativity of *α*-synuclein binding to lipid membranes. ACS Chem. Neurosci..

[68] Fusco G (2016). Structural basis of synaptic vesicle assembly promoted by *α*-synuclein. Nat. Commun..

[69] Burré J (2010). *α*-Synuclein promotes SNARE-complex assembly in vivo and in vitro. Science.

[70] Burré J, Sharma M, Südhof TC (2012). Systematic mutagenesis of *α*-synuclein reveals distinct sequence requirements for physiological and pathological activities. J. Neurosci..

[71] Dikiy I, Eliezer D (2012). Folding and misfolding of alpha-synuclein on membranes. Biochim. Biophys. Acta.

[72] Dubackic M (2021). On the cluster formation of *α*-synuclein fibrils. Front. Mol. Biosci..

[73] Souza JM, Giasson BI, Chen Q, Lee VM-Y, Ischiropoulos H (2000). Dityrosine cross-linking promotes formation of stable *α*-synuclein polymers: implication of nitrative and oxidative stress in the pathogenesis of neurodegenerative synucleinopathies. J. Biol. Chem..

[74] Mohammad-Beigi H (2015). Strong interactions with polyethylenimine-coated human serum albumin nanoparticles (PEI-HSA NPs) alter *α*-synuclein conformation and aggregation kinetics. Nanoscale.

[75] Rodrigues JC, Caseli L (2017). Incorporation of bacitracin in Langmuir films of phospholipids at the air-water interface. Thin Solid Films.

[76] Symons JL (2021). Lipidomic atlas of mammalian cell membranes reveals hierarchical variation induced by culture conditions, subcellular membranes, and cell lineages. Soft Matter.

[77] Withey P, Shen L, Graham W (1991). Fourier transform far infrared spectroscopy of a C4 bending mode. J. Chem. Phys..

[78] Ingólfsson HI (2014). Lipid organization of the plasma membrane. J. Am. Chem. Soc..

[79] Li B (2016). Sum frequency generation of interfacial lipid monolayers shows polarization dependence on experimental geometries. Langmuir.

[80] Maskarinec SA, Hannig J, Lee RC, Lee KYC (2002). Direct observation of poloxamer 188 insertion into lipid monolayers. Biophys. J..

[81] Golbek TW, Schmüser L, Rasmussen MH, Poulsen TB, Weidner T (2020). Lasalocid acid antibiotic at a membrane surface probed by sum frequency generation spectroscopy. Langmuir.

[82] Jo S, Kim T, Iyer VG, Im W (2008). CHARMM-GUI: a web-based graphical user interface for CHARMM. J. Comput. Chem..

[83] Berendsen HJ, van der Spoel D, van Drunen R (1995). GROMACS: a message-passing parallel molecular dynamics implementation. Comput. Phys. Commun..

[84] Kabsch W, Sander C (1983). Dictionary of protein secondary structure: pattern recognition of hydrogen-bonded and geometrical features. Biopolymers.

