# Supplementary material for: Elevated concentrations cause upright alpha-synuclein conformation at lipid interfaces
Source: Nat Commun. 2023 Sep 18;14:5731. doi: 10.1038/s41467-023-39843-1 (PMC10507035; doi:10.1038/s41467-023-39843-1)
Supplement: Supplementary file 1 — Supplementary Information [file 41467_2023_39843_MOESM1_ESM.pdf]

# Supplementary information for: Elevated concentrations cause upright alpha-synuclein conformation at lipid interfaces

Steven J. Roeters,*[,1,2,4] Kris Strunge,[1,4] Kasper B. Pedersen,[1,4] Thaddeus W. Golbek,[1] Mikkel Bregnhøj,[1] Yuge Zhang,[3] Yin Wang,[3] Janni Nielsen,[3] Mingdong Dong,[3] Daniel E. Otzen,[3] Birgit Schiøtt,[1,3] and Tobias Weidner*[,1]

[1]*Department of Chemistry, Aarhus University, Langelandsgade 140, 8000 Aarhus C, Denmark*

[2]*Department of Anatomy and Neurosciences, Amsterdam UMC, Vrije Universiteit, De Boelelaan 1108, 1081 HZ, Amsterdam, The Netherlands*

[3]*Interdisciplinary Nanoscience Center (iNANO), Aarhus University, Gustav Wieds Vej 14, 8000 Aarhus C, Denmark*

[4]*These authors contributed equally to the presented work.*

*E-mail: s.j.roeters@amsterdamumc.nl; weidner@chem.au.dk

# 1 Supplementary experimental and analytical details

## 1.1 Vibrational sum-frequency generation (VSFG) spectroscopy

The SFG setup has been previously described in ref. 1. In short, the SFG setup is based on a 7 Watt, 35 femtosecond laser system (Astrella, Coherent) with pulses centered at 800 nm and a repetition rate of 1 kHz. One part of the output was used to pump an optical paramet-

ric amplifier (OPA) with a non-collinear difference frequency generation (NDFG) extension (TOPAS Prime, Light Conversion) to generate broadband (FWHM ~300 $cm^{-1}$) IR pulses at 6.1 µm. A narrowband (FWHM ~15 $cm^{-1}$) visible beam was generated by guiding 1 mJ of the fundamental output through a Fabry-Perot etalon. The visible and IR beams were temporally and spatially overlapped at the sample surface. The SFG signal was focused into a spectrograph (Shamrock 303i, Andor) and detected by an EMCCD camera (Newton 971, Andor), which was operated using the *Andor SOLIS for Spectroscopy* software. VSFG spectra are recorded using an IR pulse spectral full-width-at-half-max of ~270 $cm^{-1}$ (from 1540 to 1810 $cm^{-1}$), for 5 minutes per sample scan (as an optimal acquisition time in terms of dark-noise reduction without obtaining so many cosmic rays per scan that removing them in an automated fashion becomes challenging) and for 15 seconds for the Au intensity calibration spectra in PPP, and in SSP (S-SF, S-visible, P-IR), PPP, SPS and PSP polarization combinations for the sample spectra. The various polarization combinations were recorded in a sequential manner, frequently going back to the SSP polarization combination in order to get an impression of the development of the overall signal intensity. The intensity of the chiral polarization combination, PSP, appears to be very weak as compared to the achiral polarization combinations, but was included in the analysis nonetheless, because (as can be seen in Main Text Figure 3) the spectral calculations of various frames do lead to chiral signals, and including this polarization combination will thus make us more sensitive in our quest of finding which structures are present in our experiment. The sample stage and IR beam path were flushed with nitrogen to avoid artifacts due to IR light adsorption by water vapor.

The spectra are processed using an in-house Python script as follows. First the spectra are frequency-corrected by calibrating the frequency axis with a calibrated water vapor FTIR spectrum by overlapping the water vapor peaks in the 1600-1800 $cm^{-1}$ with the sharp dips in an unpurged VSFG sample spectrum. Then, the cosmic rays are removed by detecting sudden jumps in the spectra when going to a subsequent pixel (threshold = 15 counts, which

is sharp enough to identify cosmic rays, while not modifying the VSFG signal's shape, which has a maximum intensity of ∼125 counts), and after how many pixels the photon count is again within the threshold, after which the intermediate pixels are interpolated. Then, the background spectra (recorded with blocked IR) are subtracted from the sample and the gold spectra. To compensate for minor remaining amounts of visible light due to beam pointing differences or sample evaporation during the experiments, which can create a linear offset when visible light that scatters from the trough bottom hits the detector, subsequently a linear background (fitted to the average values of the first and last 50 pixels of the 1100-2200 $cm^{-1}$ range of the CCD chip), is subtracted. In these experiments, this only yielded linear offsets of a few counts at the extremes of the CCD chip (while, again, the highest signals were ∼125 counts). Finally, the processed sample spectra are normalized using a reference spectrum obtained from gold, which has been processed with the same procedure.

## 1.2 Supplementary protein concentration notes and calculations

We note that the 'physiological' $\alpha$S concentration of ∼20 $\mu$M is based on only a single reference,[2] because this appears to be the only study that has determined this in the synapse. The number of ">1 $\mu$M" can also be found in the literature,[3–5] but we were not able to derive from which experimental data this number was derived, and furthermore it is not specific.

The determination of the LPR values of ∼0.037, 0.091 and 37, as used in the Main Text is given in the table below. The area/lipid molecule value of 55 Å$^2$ is taken from ref. 6. The factor 2 is incorporated to optimally compare our results to the literature studies, which are all bilayer studies. This way, the number of lipids exposed to the protein solution is the same for a given LPR, without affecting the physics behind the $\alpha$S-lipid interaction, because $\alpha$S is not expected to be affected by the presence of the distal lipid leaflet.[7] Using the same methodology, the higher LPR values of 0.091 and 37 are determined for the [$\alpha$S] = 20 $\mu$M and 50 nM experiments, respectively.

Supplementary Table 1: **Calculation of lipid-protein ratios. Same method was applied for the determinations of LPR = 0.091 and 37 for [$\alpha$S] = 20 $\mu$M and 50 nM, respectively.** Source data are provided in the Source Data file.

| | |
|---|---|
| **Trough:** | |
| Total area | 0.00061 $m^2$ |
| Volume | 0.002 L |
| **Protein:** | |
| Concentration | 50 $\mu$M aS |
| Moles | 1.8 x $10^{-7}$ |
| **Lipid:** | |
| Surface pressure | 13 mN/m |
| Area/lipid at a surface pressure of 13 mN/m | 55 $Å^2$ |
| Number of lipids | 1.1 x $10^{15}$ molecules |
| | 1.8 x $10^{-9}$ moles |
| **Exposed-lipid correction:** | 2 (monolayer-to-bilayer factor) |
| **LPR:** | 0.037 (lipid-to-protein ratio) |
| **PLR:** | 27 (protein-to-lipid ratio) |

## 1.3 Supplementary details on the molecular dynamics simulation setup

Eight systems of $\alpha$S in contact with, or in the presence of a DPPG monolayer were created using the CHARMM-GUI webserver.[8]

In the first three systems, the starting conformations of $\alpha$S adopt a kinked $\alpha$-helix 'horseshoe' conformation (PDB: 2KKW [http://doi.org/10.2210/pdb2KKW/pdb] (SLAS-micelle bound $\alpha$S )[9]): system 1 (2KKW, model 1), system 2 (2KKW, model 11), and system 6 (2KKW, model 26). System 1 and 2 are rotated 180 degrees with respect to each other, such that both "sides" of the $\alpha$-helix horseshoe are interfaced with the monolayer. In system 6 the horseshoe was rotated 90 degrees, compared to system 1 and 2, and is only in contact with lipids via the N-terminus.

Additionally, we created two systems where $\alpha$S adopts an upright conformation protruding away from the surface (system 4, 5), and one (system 7) lying flat on the membrane with several kinks, compared to PDB: 2KKW [http://doi.org/10.2210/pdb2KKW/pdb] (SLAS-micelle bound $\alpha$S ). The $\alpha$S structures used in system 4, 5 and 7 were obtained from prelim-

inary simulations of $\alpha$S on DPPG bilayers and were found to match the low LPR ensemble (system 4,5) and the high LPR ensemble (system 7). These structures were included to diversify sampling of the $\alpha$S conformational landscape.

Furthermore, two control systems were created: one without secondary structure (i.e. $\alpha$S is completely disordered), in contact with the monolayer (system 3), and one where $\alpha$S was placed in the vacuum above the monolayer (system 8). The starting structure of system 8 moves from the vacuum to the backside (tails) of the monolayer within ~500 ps and displaces lipids during the 150 ns trajectory (Figure 7). All starting conformations are depicted in Figure 6.

All $\alpha$S residues were built in standard protonation states (pH 7.4) with H50 being neutral and in the $\delta$-tautomer. Protein termini were uncapped and charged. The monolayer systems consist of a water region (100,000 water molecules) bordered by two DPPG monolayers (250 lipids in each, head groups facing the water). The monolayer was built using an area per lipid of 55 $Å^2$, mimicking the liquid-condensed (LC) monolayer phase. A large vacuum region separates the DPPG monolayers. The water region contains 768 $Na^+$, and 259 $Cl^-$ ions (0.15 M NaCl). The box size of the systems are ~11.7 nm x 11.7 nm x 81.5 nm.

Molecular dynamics simulations were performed using GROMACS[10] v2021.4 on Nvidia V100 GPUs at the Centre for Scientific Computing Aarhus (CSCAA). We use a combination of the DES-amber force field[11] for protein, water, and ions, and the Slipid forcefield[12–14] for lipids. A non-bonded truncation cutoff of 1 nm was used and the particle mesh Ewald[15] method was used for long-range electrostatic summation. The systems were first minimized and equilibrated in two steps: (1) 250 ps (1 fs timesteps) in the NVT ensemble using the v-rescaling thermostat[16] at 296.15 K (the temperature in our laser lab) with position restraints on the DPPG molecules and the protein backbone; (2) 10 ns (2 fs timesteps) using the same settings and hydrogen bonds restrained using LINCS but not restraints on DPPG. Production runs of the eight systems were simulated for 150 ns with two repeats in each simulation using the same setting as step (2) but without position restraints on the protein.

Velocities were resampled from the Maxwell-Boltzmann distribution for each repeat. Frames were saved every 50 ps.

We note that one should be careful combining force fields. However, Slipids were developed to be compatible with the AMBER99SB-ILDN force field branch which is also the foundation of the DES-amber force field, and thus this force field combination is currently the most consistent option for simulation of IDPs and lipids. However, since the LJ interactions between TIP4P-D (water model in DES-amber) and the DES-amber protein parameters are increased, compared to other protein force fields and water models, e.g. TIP4P+AMBER99SB-ILDN, this could shift the relative interactions between protein and lipids. We stress that our MD simulations are used as a way to generate realistic hypothetical conformations of $\alpha$S near a DPPG monolayer, and that the fit RSS of the VSFG spectral calculation is used to extract an ensemble consistent with the VSFG experiment.

## 1.4 Spectral calculations

The spectral calculations are based on a excitonic Hamiltonian approach developed first described in ref. 17. Because they are based on the 16 150 ns MD trajectories, we performed the calculations for a total of 48.016 different frames, with a varying protein structure and orientation in each frame. Because we did not find an improvement of the spectral match when we averaged over multiple frames (see Table S3), all presented spectral calculation are performed by directly obtaining the eigenmodes from each frame, by evaluating the excitonic Hamiltonian model. The residual sum-of-squares (RSS) between the experimental and calculated spectra was used to find the frames that describe the experimental response best.

In the spectral calculations (also known as frequency mapping[18]), the exact spectral simulation parameters that are used can be very important for the outcome. The nearest-neighbor coupling was described using a dihedral-angle parameterized map of *ab initio* B3LYP/6-31G+(d) coupling from N-Methyldacetamide (NMA) deuterated at the amide group's N

atom. Transition dipole momenta were based on a map of Mulliken charges from an *ab initio* B3LYP/6-31G+(d) calculation. The frequency shift applied for the five proline residues in $\alpha$S was $\delta\omega_{\text{proline}} = 19\,\text{cm}^{-1}$. The refractive indices used to describe the local-field corrections are listed in Table 2 for the bulk-air phase, bulk-water phase and the refractive index of the thin interfacial lipid/protein film. The interfacial refractive index is important in the description of $L_{zz}(\omega_j)$ in the Fresnel factor equations.[19,20] It is a weak point in determining the local mode corrections, since it is particularly hard to determine with a consistent and independent approach, but the frequently-employed estimation of Zhuang et al.[19] has resulted in consistent molecular pictures in many studies.[20,21]

Supplementary Table 2: **Refractive indices used for estimation of the local field corrections.** Water data from ref.[22] and the interfacial refractive index from ref.[19] Source data are provided in the Source Data file.

| | $\omega_{SF}$ | $\omega_{VIS}$ | $\omega_{IR}$ |
|---|---|---|---|
| $n_1$ (air) | 1 | 1 | 1 |
| $n_2$ (water) | 1.331 | 1.329 | 1.315 |
| $n'$ | 1.18 | 1.18 | 1.18 |

### 1.4.1 Calibration of the spectral calculations with LK$\alpha$14

To calibrate the local-mode frequency parameters (the frequency of the uncoupled local modes and influence of hydrogenbonding on the local-mode frequency, estimated by a linear correlation with the C=O bond length[23,24]), we compare experimental and calculated spectra of the LK$\alpha$14 peptide.[25] LK$\alpha$14 is a short peptide of leucine and lysine residues (Ac-LKKLLKLLKKLLKL). It is specifically engineered to fold into a stable $\alpha$-helical structure at a hydrophilic/hydrophobic interface. LK$\alpha$14 is simulated for 300 ns in 3 replications on a water-hydrofobic (PG lipid-tails) interface. The experimental data is taken from ref. 25 of LK$\alpha$14 at a hydrophobic polystyrene interface in water. The reflections of the sum-frequency–, visible– and infrared– beams, as well as the different VIS-wavelength ($\lambda = 532$ nm) beams were compensated for with the appropriate local-field corrections. By optimizing the spectral parameters of the calculations to optimally match the SSP and PPP

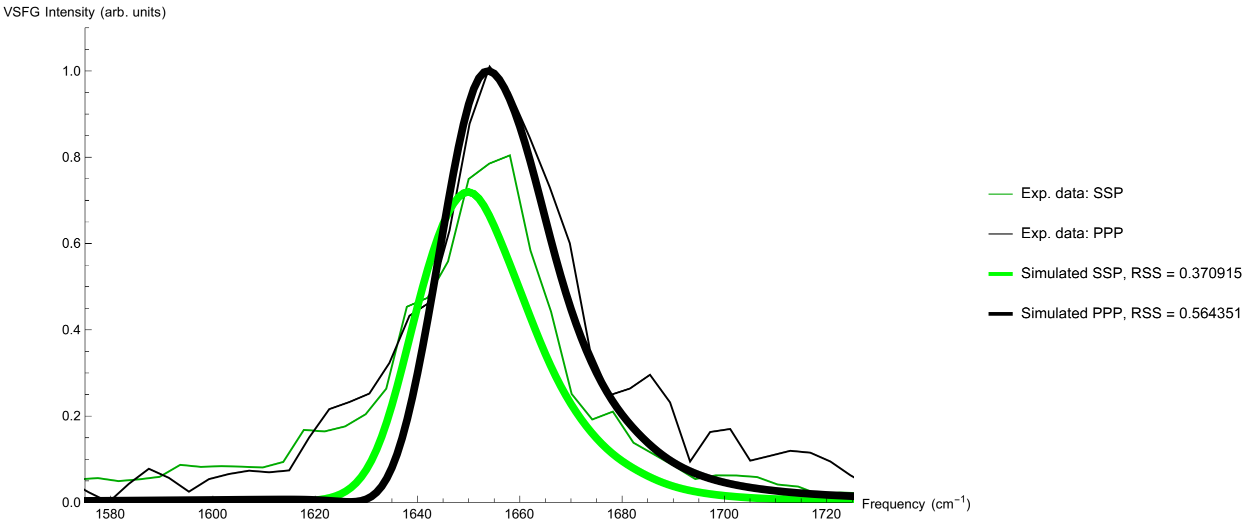


Supplementary Figure 1: **Experimental (rugged) and calculated (smooth) spectra of LK$\alpha$14, used to calibrate the local-mode frequency parameters of the spectral calculations.** Source data are provided in the Source Data file.

spectra experiments, we found the slope parameter of the hydrogen-bonding effect on the local-mode frequency to be $\delta\omega_{HB} = 400\,\text{cm}^{-1}\text{Å}^{-1} r_{CO}$ and isolated local-mode frequency $\Omega^0$ was $1655\,\text{cm}^{-1}$ (see Figure 1). The experimental LK$\alpha$14 data is recorded with a picosecond VSFG scanning setup in Seattle, as opposed to the $\alpha$-synuclein data recorded in Aarhus from the femtosecond Astrella laser in the Weidner Lab. The linewidth for the two setups is different, so this parameter was therefore optimized for the $\alpha$-synuclein spectrum. However, the best protein structure was found to be independent of the linewidth, within a range around the expected experimental linewidth (see Table S3).

### 1.4.2 Supplementary discussion of spectral match

The resulting spectra with the lowest RSS (see Figure 3) closely match the relative SSP and PPP intensities, as well as their lineshape. The SPS match is slightly less good, which can be understood from the fact that selection based on the total RSS of all polarization combinations results in a higher sensitivity to stronger polarization combinations. The lack of any (chiral) PSP intensity is reproduced well, and the transparent band that indicates the bandwidth of the spectra calculated for the frames within the ensembles shows that including this polarization combination filters out structures that have a chiral response, contrary to the experimental observations. It is interesting to note that most deviations within the transparent band are in the 1660-1680 cm-1 region, where turns are thought to absorb IR light.[26] Also based on other recent results obtained with this Hamiltonian approach, we think that this is due to force-field imperfections that lead to too-steep turns. Happily, when taking the average of the structures in the ensemble the effect averages out, or at least has a negligible effect on the spectral lineshapes.

#### 1.4.3 Supplementary discussion of spectral-calculation results for the MD simulations

A more detailed comparison of the molecular dynamics(MD) simulations and the spectral-calculation derived RSS values reveals that, for all the trajectories, the kinked $\alpha$-helical starting structure partly unfolds, resulting in shorter helical segments linked by disordered regions (see Figures 2(C) and 12). The $\alpha$-helical contents (quantified by the DSSP algorithm[27]) generally decreases throughout the 150-ns trajectories, in some cases $\sim$20-30%. Interestingly, there is no clear correlation between the obtained RSS values and the helicity, indicating — as expected — that the VSFG response is sensitive to other structural aspects besides the secondary structure alone.

Furthermore, although the difference is not large, from Figure 10, it appears that – while most segments have a very similar secondary structure distribution, there is a distinct subset of structures with a helicity of $\sim$30% at low concentrations, which is absent in the high concentration ensemble. The capability to resolve such detailed structural differences is a valuable feature that the frame-selection method allows.

#### 1.4.4 Supplementary discussion of spectral calculations for literature models

For 2KKW [http://doi.org/10.2210/pdb2KKW/pdb] (SLAS-micelle bound $\alpha$S), for both LPRs, all available models within the PDB entry were tried in order to find the model with the best match. This resulted in model 26 for the low-LPR dataset, and in model 18 for the high-LPR dataset. For the structures (of $\alpha$S fragment (residues 9-89) bound to 7:3 POPC/POPS small unilamellar vesicles[28]) provided by the Langen group, only the first structure out the identified ensemble was analyzed, as all of the structures were structurally very similar. Although a small part of the N-terminus and the full C-terminus is missing, we expect that this will only minutely influence the result, as random-coiled structures hardly contribute to VSFG signals.[20] For the low-LPR dataset, the best 2KKW orientation results in a reasonably extended protein orientation, and also the degree of helicity is not very

different in the best-matching MD-simulation frames, which explains why the RSS of this structure and orientation is relatively low. Lorentzian widths of both 8 and 12 $cm^{-1}$ have been tried, finding that a width of 12 $cm^{-1}$ gave the best match. Most importantly, for the low-LPR dataset, the orientations reported in the associated publications do not appear to be consistent with our experimental results at all, given the very large RSS values at y rotation = $\sim 90°$ for the horseshoe structure[9] and at both x and y rotation = 90° for the Langen structure,[28] while for the high-LPR dataset these orientations actually lead to a close match, which corroborates the similar orientations derived with the frame-selection method described in the main text, thus benchmarking this novel approach.

## 1.5 Atomic-force microscopy

The AFM samples have been prepared by performing a Langmuir-Schaefer deposition of the sample surface on freshly-cleaved mica, followed by drying for 10 min at the ambient environment. Subsequently, the sample on mica was imaged.

The AFM imaging was conducted on the Multimode® VIII AFM (Bruker, CA) in a Peakforce tapping mode. An silicon nitride probe Scanasyst-Air with the spring constant of 0.4 N/m was used under the ambient environment. The setpoint is less than 200 pN in order to protect sample. The raw images were processed by the software SPIP™(Scanning Probe Image Processor) software package (Image Metrology, Denmark).

# 2 Supplementary calculation results

## 2.1 RSS values for various Lorentzian widths and frame-averaging sizes

Supplementary Table 3: **Table of key parameters from the investigation of the dependency of the best matching $\alpha$-synuclein structure on the broadening parameter $\Gamma$.** "Best structure" refers to the structure with the lowest RSS value. The half-width-at-half-max (HWHM) $\Gamma = 8\,\text{cm}^{-1}$ is used as it is the expected experimental linewidth of the visible pulse coming out of the etalon, but it is also observed that the conclusion from the calculation is stable within $\sim 2\,\text{cm}^{-1}$. The structure in frame 2599 is shown in main text figure 2(C). The best matching structures from $\Gamma = 6 - 12\,\text{cm}^{-1}$ are all very similar in structure and orientation. Source data are provided in the Source Data file.

| $\Gamma$ [$\text{cm}^{-1}$] | Lowest RSS | MD frame of best structure | RSS of frame 2599 |
|---|---|---|---|
| 6 | 1.04 | 2423 | 1.29 |
| **8** | **1.16** | **2599** | **1.16** |
| 10 | 1.10 | 2599 | 1.10 |
| 12 | 1.08 | 7710 | 1.53 |
| 16 | 1.32 | 397 | 5.45 |
| 20 | 1.62 | 526 | 5.22 |

Supplementary Table 4: **RSS as a function of the number of successive frames over which the local-mode frequencies (that are a function of the hydrogenbonds) are averaged, for each of the production runs.**

| Frames per segment | Min RSS s1_r1 | Min RSS s1_r2 | Min RSS s1_r3 | Min RSS s2_r1 | Min RSS s2_r2 | Min RSS s2_r3 |
|---|---|---|---|---|---|---|
| 1 | 1.16 | 1.25 | **1.11** | 1.17 | 1.32 | 1.44 |
| 10 | **1.11** | **1.14** | 1.22 | **0.98** | **1.21** | 1.28 |
| 100 | 1.25 | 1.23 | 1.29 | 1.05 | 1.46 | **1.23** |

## 3 Supplementary experimental results

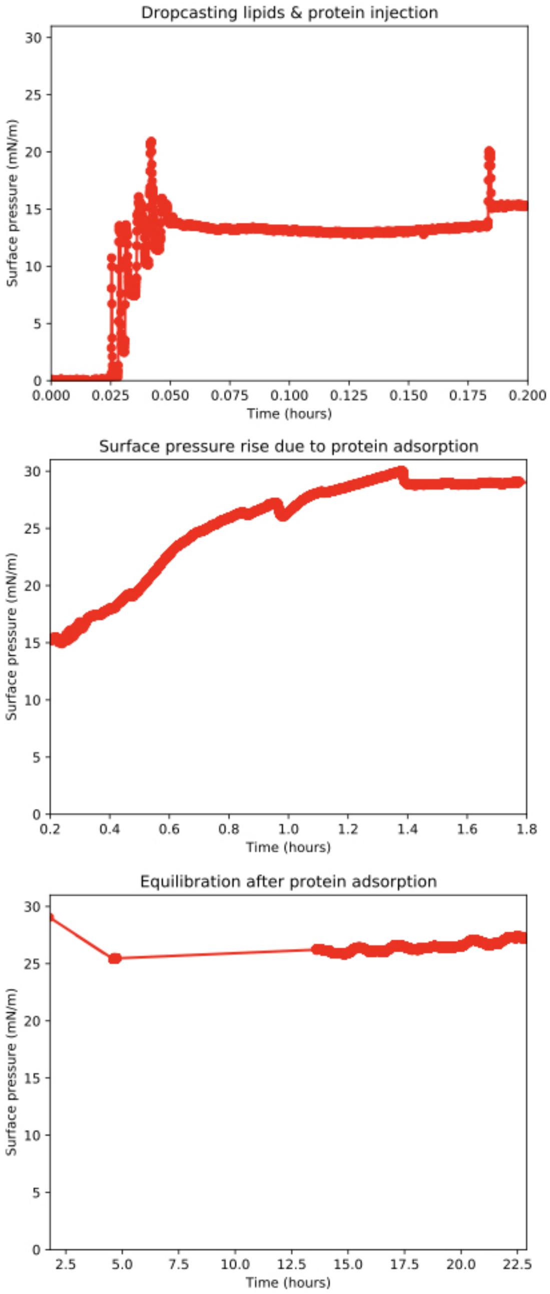


Supplementary Figure 2: **Surface-pressure as a function of incubation time at 50 $\mu$M $\alpha$S concentration.** At t = 0.025 hr, the lipid monolayer was prepared by dropcasting a chloroform-based 1 mg/mL DPPG solution on the air-water interface. After equilibration for ∼10 minutes, the high-concentration (245 $\mu$M) $\alpha$S solution is injected, after which the VSFG experiments were started. The qualitative response of a fast initial surface-pressure rise and subsequent relaxation has been reproduced 4 times; in a similar fashion for LPR values of ∼0.04, ∼0.1 and ∼37. Source data are provided in the Source Data file.

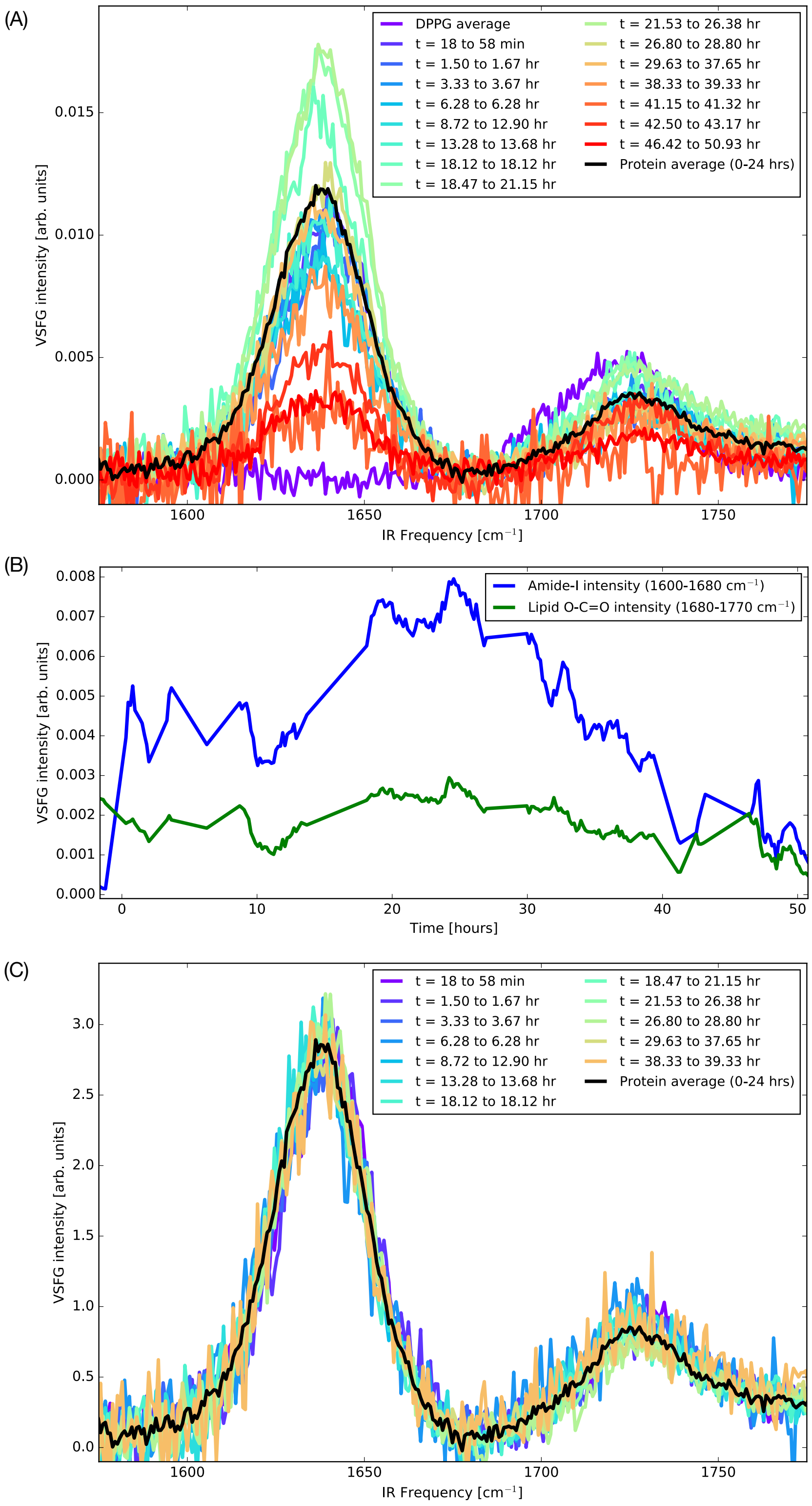


Supplementary Figure 3: **Intensity of the SSP polarization combination as a function of time for low LPR (∼0.037; this page) and high LPR (∼37; next page).** (A) non-normalized, (B) amide-I and lipid-ester band intensity versus time, and (C) normalized spectra (the weak last 3 spectra from the time series are left out for clarity, but also those have an identical lineshape). Source data are provided in the Source Data file.

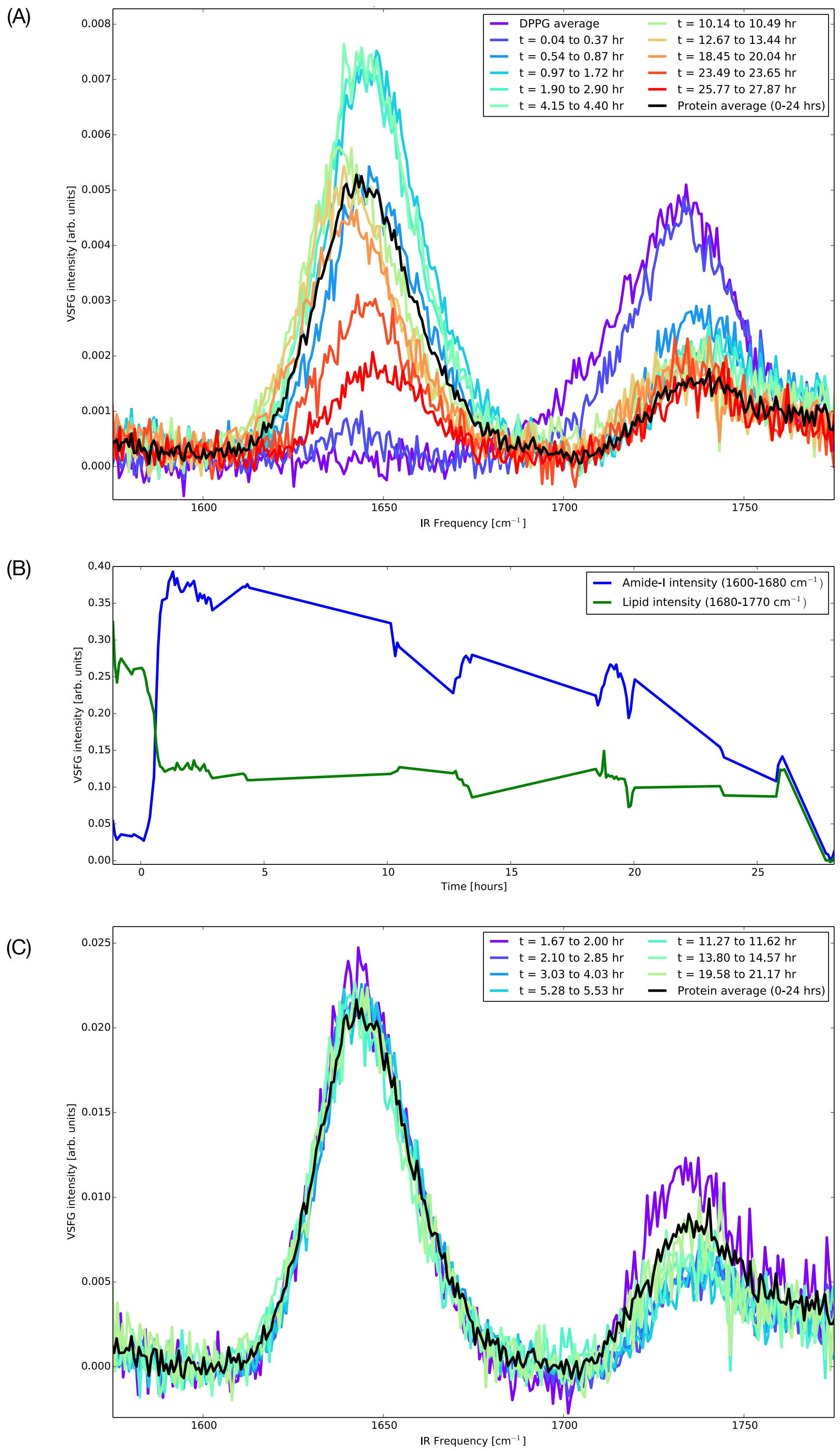
(A)
DPPG average
t = 0.04 to 0.37 hr
t = 0.54 to 0.87 hr
t = 0.97 to 1.72 hr
t = 1.90 to 2.90 hr
t = 4.15 to 4.40 hr
t = 10.14 to 10.49 hr
t = 12.67 to 13.44 hr
t = 18.45 to 20.04 hr
t = 23.49 to 23.65 hr
t = 25.77 to 27.87 hr
Protein average (0-24 hrs)
VSFG intensity [arb. units]
0.000
0.001
0.002
0.003
0.004
0.005
0.006
0.007
0.008
1600
1650
1700
1750
IR Frequency [cm$^{-1}$]
(B)
Amide-I intensity (1600-1680 cm$^{-1}$)
Lipid intensity (1680-1770 cm$^{-1}$)
VSFG intensity [arb. units]
0.00
0.05
0.10
0.15
0.20
0.25
0.30
0.35
0.40
0
5
10
15
20
25
Time [hours]
(C)
t = 1.67 to 2.00 hr
t = 2.10 to 2.85 hr
t = 3.03 to 4.03 hr
t = 5.28 to 5.53 hr
t = 11.27 to 11.62 hr
t = 13.80 to 14.57 hr
t = 19.58 to 21.17 hr
Protein average (0-24 hrs)
VSFG intensity [arb. units]
0.000
0.005
0.010
0.015
0.020
0.025
1600
1650
1700
1750
IR Frequency [cm$^{-1}$]

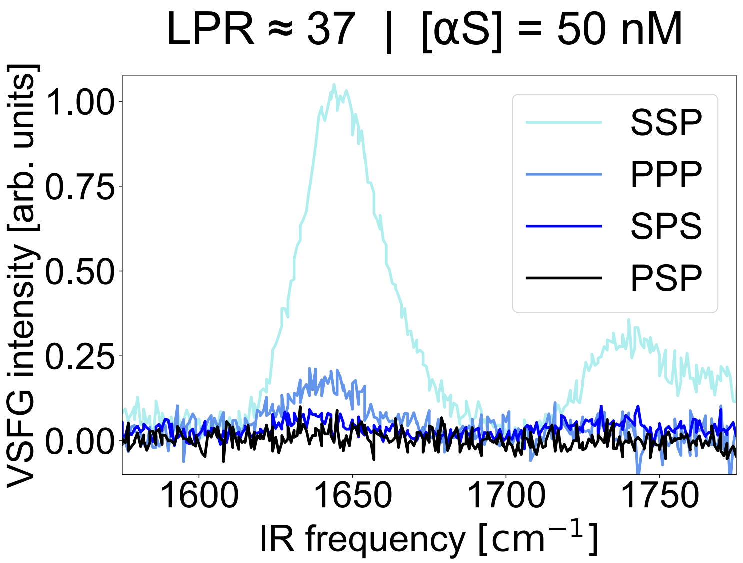


Supplementary Figure 4: **Average of stable ∼15 hours of experimental VSFG data of 50 nM $\alpha$S concentration incubated under a DPPG monolayer, at a surface pressure of 30 mN/m (obtained after injection of $\alpha$S in the subphase).** Source data are provided in the Source Data file.

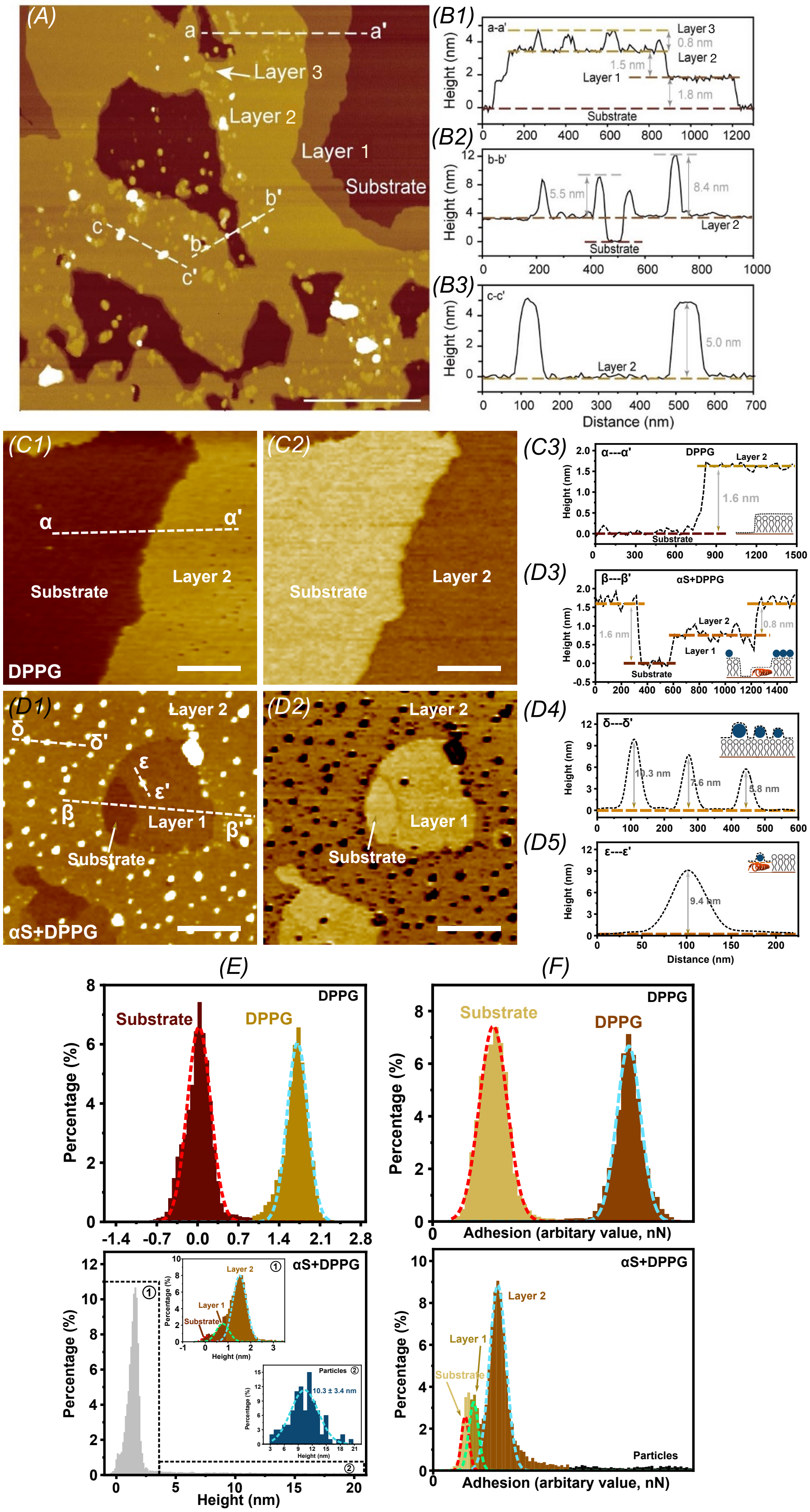
(A)
a
a'
Layer 3
Layer 2
Layer 1
Substrate
b'
c
b
c'
(B1)
a-a'
Layer 3
0.8 nm
Layer 2
1.5 nm
Layer 1
1.8 nm
Substrate
Height (nm)
(B2)
b-b'
5.5 nm
8.4 nm
Layer 2
Substrate
(B3)
c-c'
5.0 nm
Layer 2
Distance (nm)
(C1)
α
α'
Substrate
Layer 2
DPPG
(C2)
Substrate
Layer 2
(C3)
α---α'
DPPG
Layer 2
1.6 nm
Substrate
(D3)
β---β'
αS+DPPG
Layer 2
Layer 1
1.6 nm
0.8 nm
Substrate
(D1)
δ
δ'
ε
ε'
Layer 2
β
β'
Layer 1
Substrate
αS+DPPG
(D2)
Layer 2
Layer 1
Substrate
(D4)
δ---δ'
10.3 nm
7.6 nm
5.8 nm
(D5)
ε---ε'
9.4 nm
Distance (nm)
(E)
DPPG
Substrate
DPPG
Percentage (%)
αS+DPPG
Layer 2
Layer 1
Substrate
Height (nm)
Particles
10.3 ± 3.4 nm
Height (nm)
(F)
DPPG
Substrate
DPPG
Adhesion (arbitary value, nN)
αS+DPPG
Layer 2
Layer 1
Substrate
Particles
Adhesion (arbitary value, nN)

Supplementary Figure 5 *(preceding page)*: **AFM images taken of regions with defects of Langmuir-deposited interfaces (on freshly-cleaved mica), which show that there are no fibrillar species present at the water–lipid interface.** (A) Height map of a DPPG monolayer that has been incubated for 5-hours with 50 $\mu$M of $\alpha$S (LPR = 0.037), indicating that at these incubation times and concentrations the proteins form a $\sim$1.5 nm thick monolayer on top of an $\sim$1.8 nm DPPG layer (consistent with the lipid lengths found in the MD simulations). On top of these layers, oligomeric species are observed, which might either be an artifact from the drying step in the sample preparation, or from protein aggregation during the experiment. (B1-3) Three associated height profiles. (C1,C2) Height and adhesion maps of a pure DPPG monolayer. (D1,D2) Similar, but for a DPPG monolayer incubated for 24 hours with an $\alpha$S solution at LPR = 37. As can be seen in the height profiles depicted in (C3) and (D3-5), the lipid monolayer is $\sim$1.6 nm high, the monomeric protein layer is $\sim$0.8 nm high (consistent with the diameter of an $\alpha$-helix), while the oligomers (mostly to be found on the lipid layer, but there are also some found on the protein layer) are $\sim$10 nm high. It is interesting to note that the largest oligomers are found on the edge of the areas where the lipids have been displaced by the proteins. (E,F) Height and adhesion histograms of the AFM image for DPPG (top) and $\alpha$S -incubated DPPG (bottom) corroborate the layer assignment through the different adhesion values for the lipid and the protein layers. All scale bars are 1 $\mu$m. Source data are provided in the Source Data file.

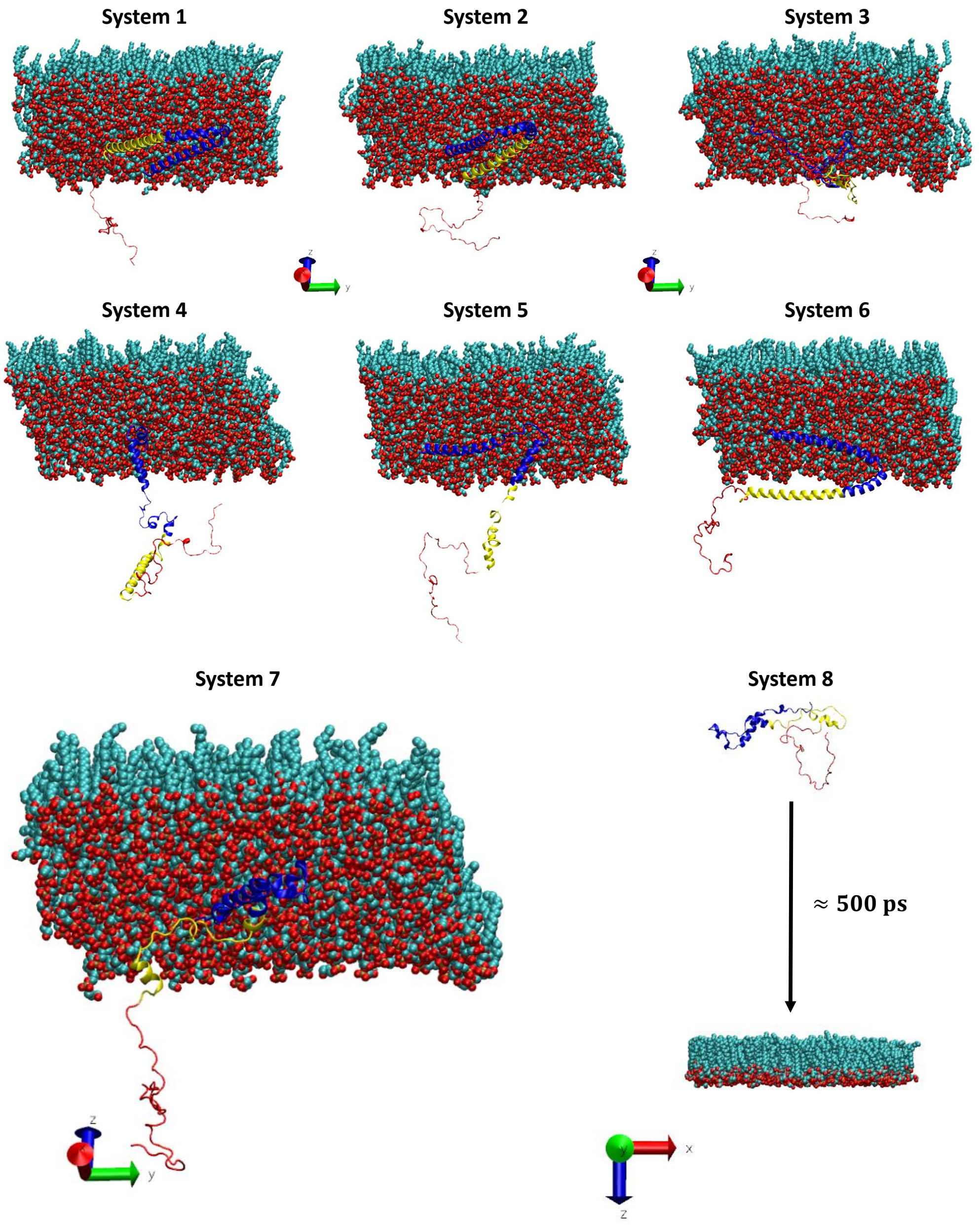


Supplementary Figure 6: **Starting conformations of the eight monolayer systems.** (System 1, 2, 6) Kinked $\alpha$-helix 'horseshoe' conformation (PDB: 2KKW [http://doi.org/10.2210/pdb2KKW/pdb] (SLAS-micelle bound $\alpha$S )[9]) in different orientations. (system 4,5) Upright conformation protruding away from the surface. (system 3) Completely disordered in contact with the monolayer. (system 7) Flat lying conformation on the membrane with several kinks. (system 8) $\alpha$S placed in the vacuum above the monolayer which goes to the backside and penetrates the monolayer.

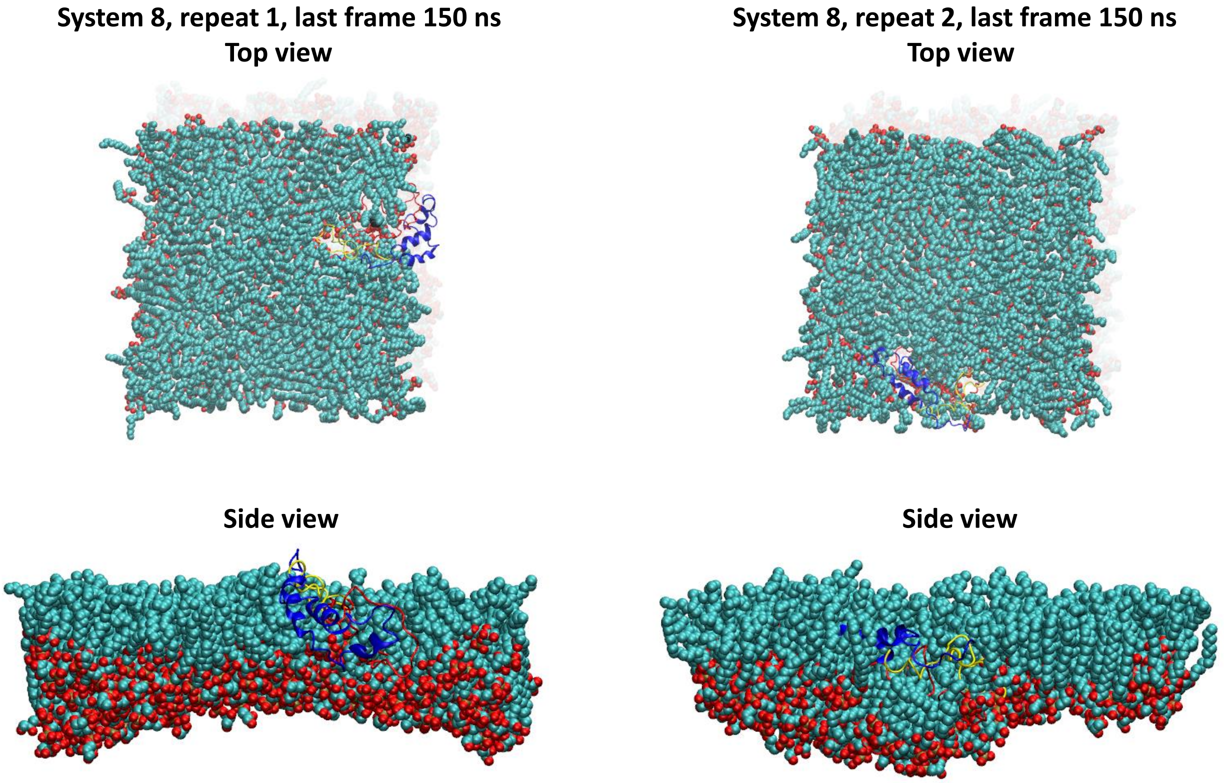


Supplementary Figure 7: **Last frames of both runs that started with starting structure 8 (see Figure S6), i.e., where the $\alpha$S molecule is placed on the lipid-tail side of the monolayer.** During these trajectories, $\alpha$S displaces lipids and penetrates the monolayer.

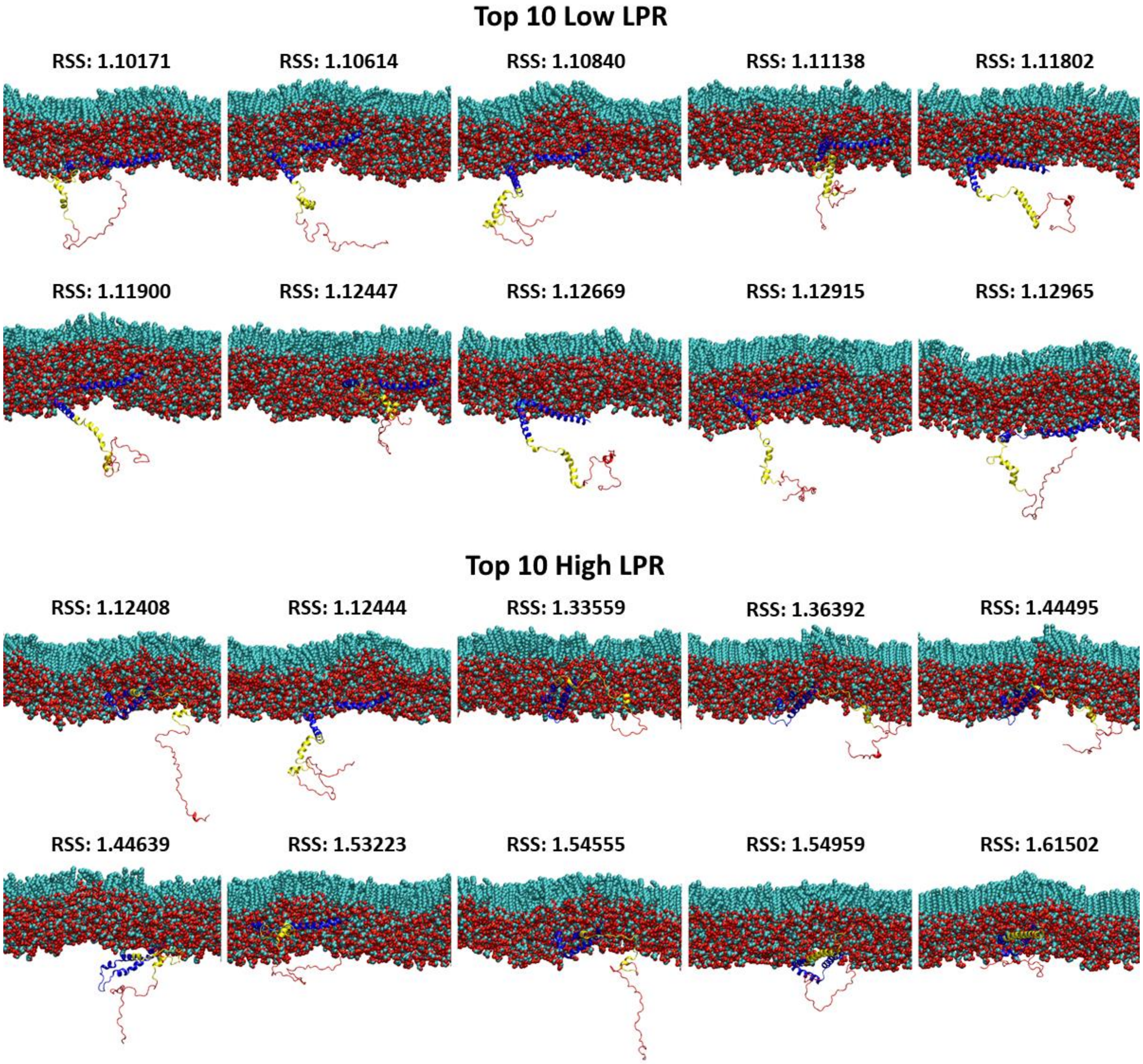


Supplementary Figure 8: **Overview of the 10 best-matching frames for the low-LPR (row 1-2) and high-LPR (row 3-4) experiments, as assessed by their RSS (the residual sum-of-squares between the calculated and experimental spectra).** Comparison of the two ensembles reveal a clear trend of upright conformations in the low LPR top 10 and flat-lying structures in the high-LPR top 10.

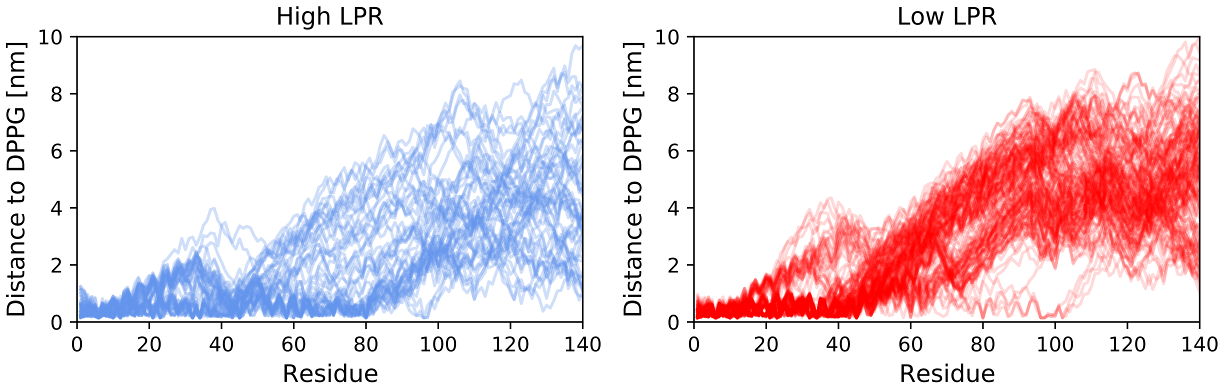


Supplementary Figure 9: **Distances to the nearest DPPG molecules for each residue of the conformations within the high-LPR and low-LPR ensembles.**

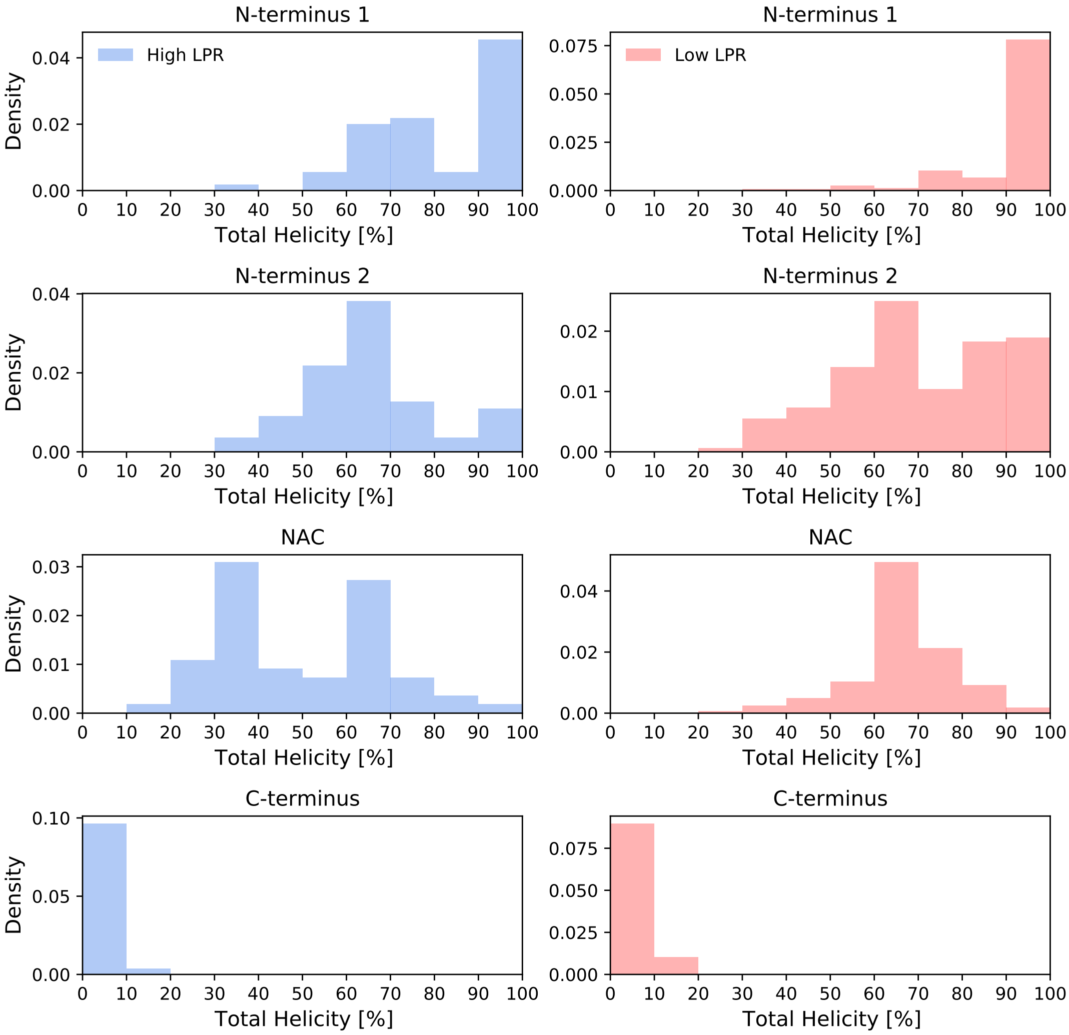


Supplementary Figure 10: **Helical contents of the high- and low-LPR ensembles, divided into 4 protein regions: the N-terminus 1 (residue 1–30), the N-terminus 2 (residue 31–60), NAC region (residue 61–95) and C-terminus (residue 96–140).**

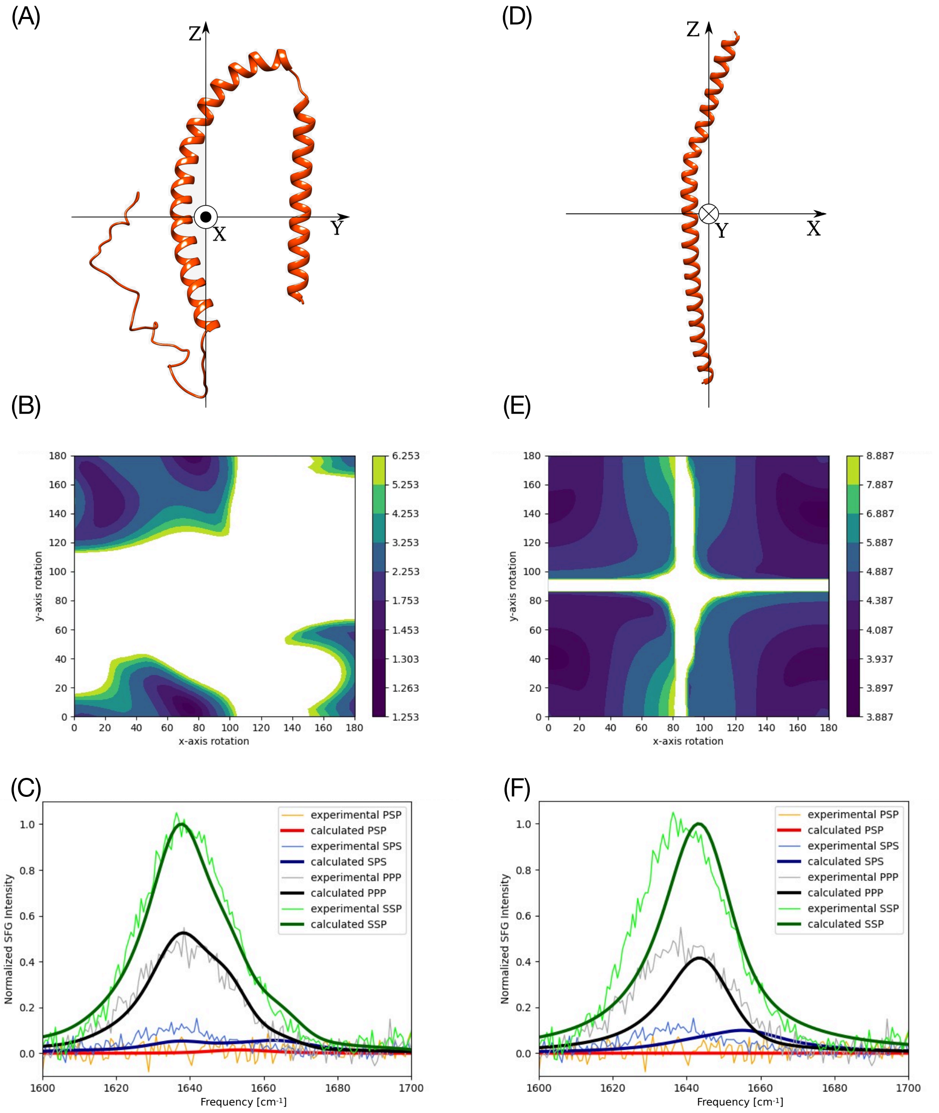


Supplementary Figure 11: **Structures and best-matching calculated VSFG spectra of two literature structures: low LPR (this page) and high LPR (next page).** Left column: (A) model 26 of PDB 2KKW [http://doi.org/10.2210/pdb2KKW/pdb] (SLAS-micelle bound $\alpha$S ) with (x,y)-axis rotations of (0°,0°), (B) the 2D-RSS plot as a function of the two rotation angles with a 4° resolution, and (C) the experimental and calculated spectra with a lowest RSS of 1.25 for an (x, y) rotation of (73°, 175°). Right column: (D) model 1 of the structural ensemble provided the Langen group, rotated over the (x,y)-axes by (0°,0°), (E) the associated 2D-RSS plot as a function of the two rotation angles with a 4° resolution, and (F) the experimental and calculated spectra with a lowest RSS of 3.89 for an x, y rotation of (175°, 40°). Same plots are shown on the next page, but for the match with the experimental high-LPR data, and with (A, B) the best orientations obtained for the best-performing model (#18) of PDB 2KKW (RSS = 1.540 for an (x, y) rotation of (61°, 65°)) and for model 1 of the Langen structure (RSS = 1.201 for an (x, y) rotation of (94°, 77°)), respectively. These results are discussed in detail in the 'Spectral calculations' section in the SI. Source data are provided in the Source Data file.

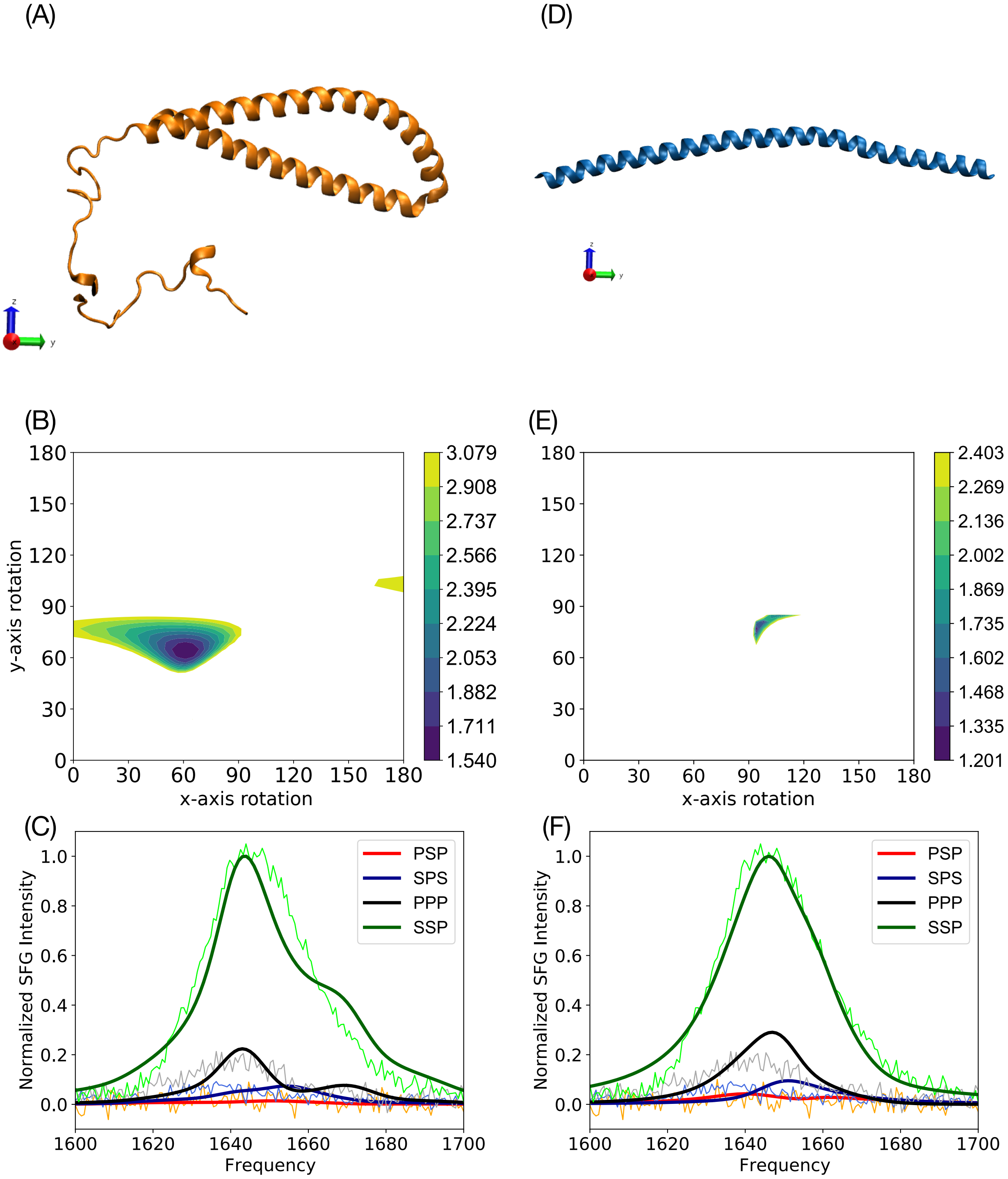

(A)
(D)
(B)
(E)
(C)
(F)
y-axis rotation
x-axis rotation
3.079
2.908
2.737
2.566
2.395
2.224
2.053
1.882
1.711
1.540
2.403
2.269
2.136
2.002
1.869
1.735
1.602
1.468
1.335
1.201
Normalized SFG Intensity
Frequency
PSP
SPS
PPP
SSP

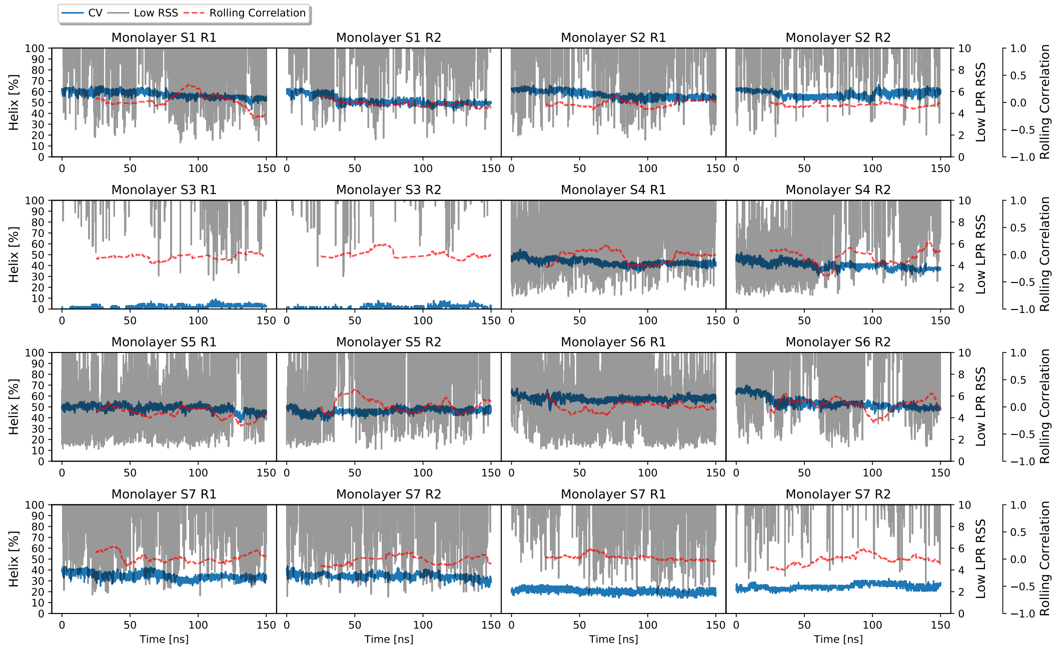


Supplementary Figure 12: **RSS values for low- (this page) and high- (next page) LPR plotted together with the 'collective variable' (CV) that indicates the fraction of residues found in a helix as a function simulation time for the 16 reproduction runs, along with the rolling correlation with a 25 ns window.** The helicity is calculated with the simplified DSSP scheme, MDTraj v1.9.4. Source data are provided in the Source Data file.

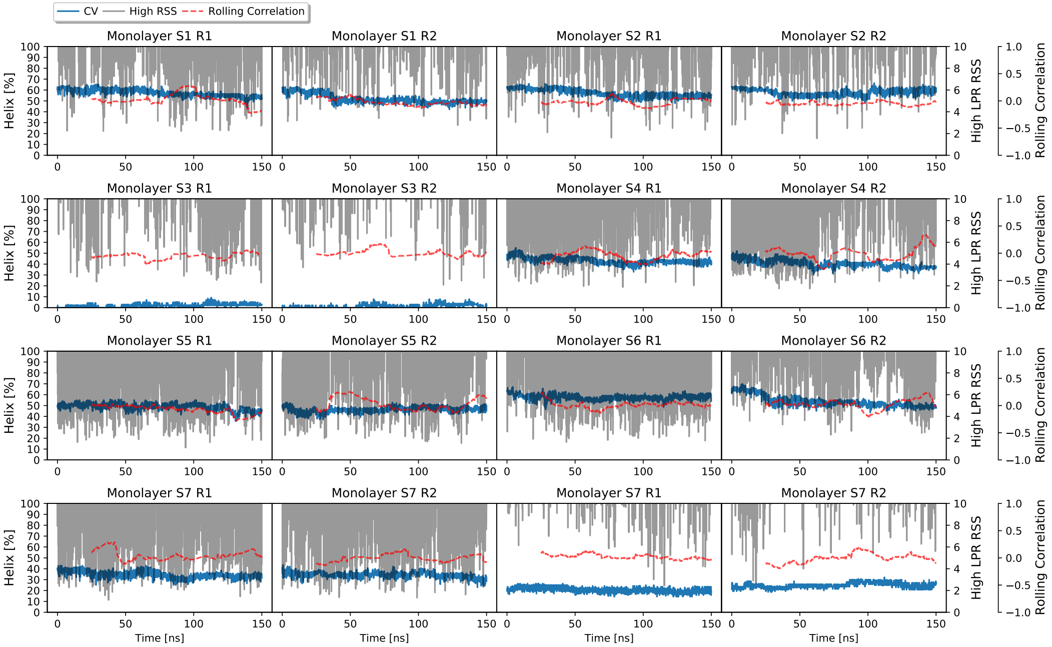

CV
High RSS
Rolling Correlation
Monolayer S1 R1
Monolayer S1 R2
Monolayer S2 R1
Monolayer S2 R2
Monolayer S3 R1
Monolayer S3 R2
Monolayer S4 R1
Monolayer S4 R2
Monolayer S5 R1
Monolayer S5 R2
Monolayer S6 R1
Monolayer S6 R2
Monolayer S7 R1
Monolayer S7 R2
Monolayer S7 R1
Monolayer S7 R2
Helix [%]
High LPR RSS
Rolling Correlation
Time [ns]

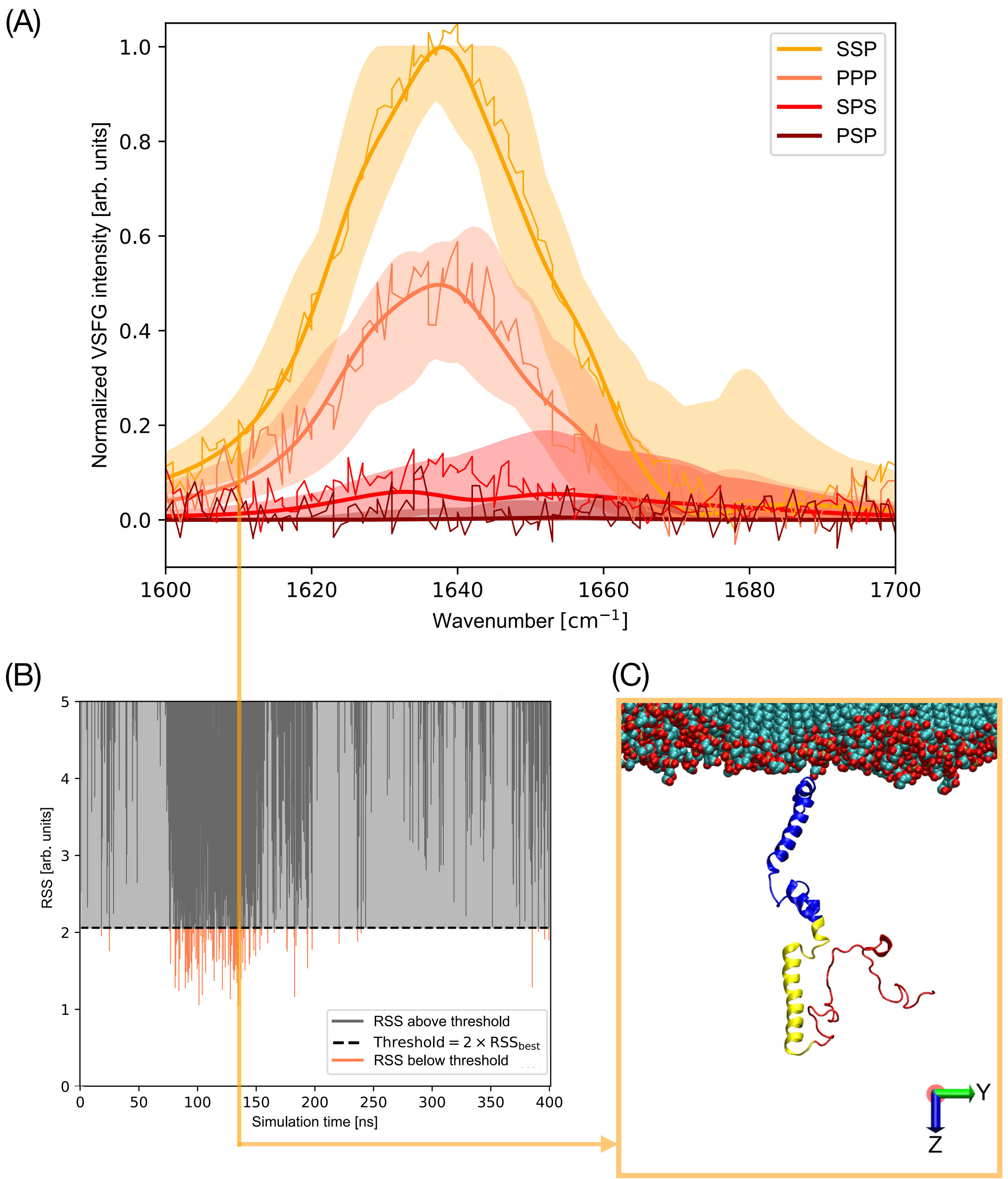


Supplementary Figure 13: **Modified, but comparable figure to the low-LPR (50 $\mu$M) depicted in Figure 3(E) in the main text, but for a reproduction dataset where the 100 kDa filtration step was left out.** The threshold used in (B) is twice the RSS of the best matching frame (as opposed to the threshold used in the main text that was set such that the standard deviation of the spectra of the ensembles matched the experimental standard deviation), and the structure depicted in (C) belongs to this best-matching frame. The similarity between the two analyses may corroborate the finding that the oligomers (which are expected to be filtered out by the 100 kDa filters) are VSFG silent, but the fact that similar kinetics were observed indicates also that there are probably few oligomers formed in the $\alpha$S production process. Furthermore, the similarity between the selected structures in the main text analysis and best-matching structure depicted in (C) substantiates the robustness of the presented approach. Source data are provided in the Source Data file.

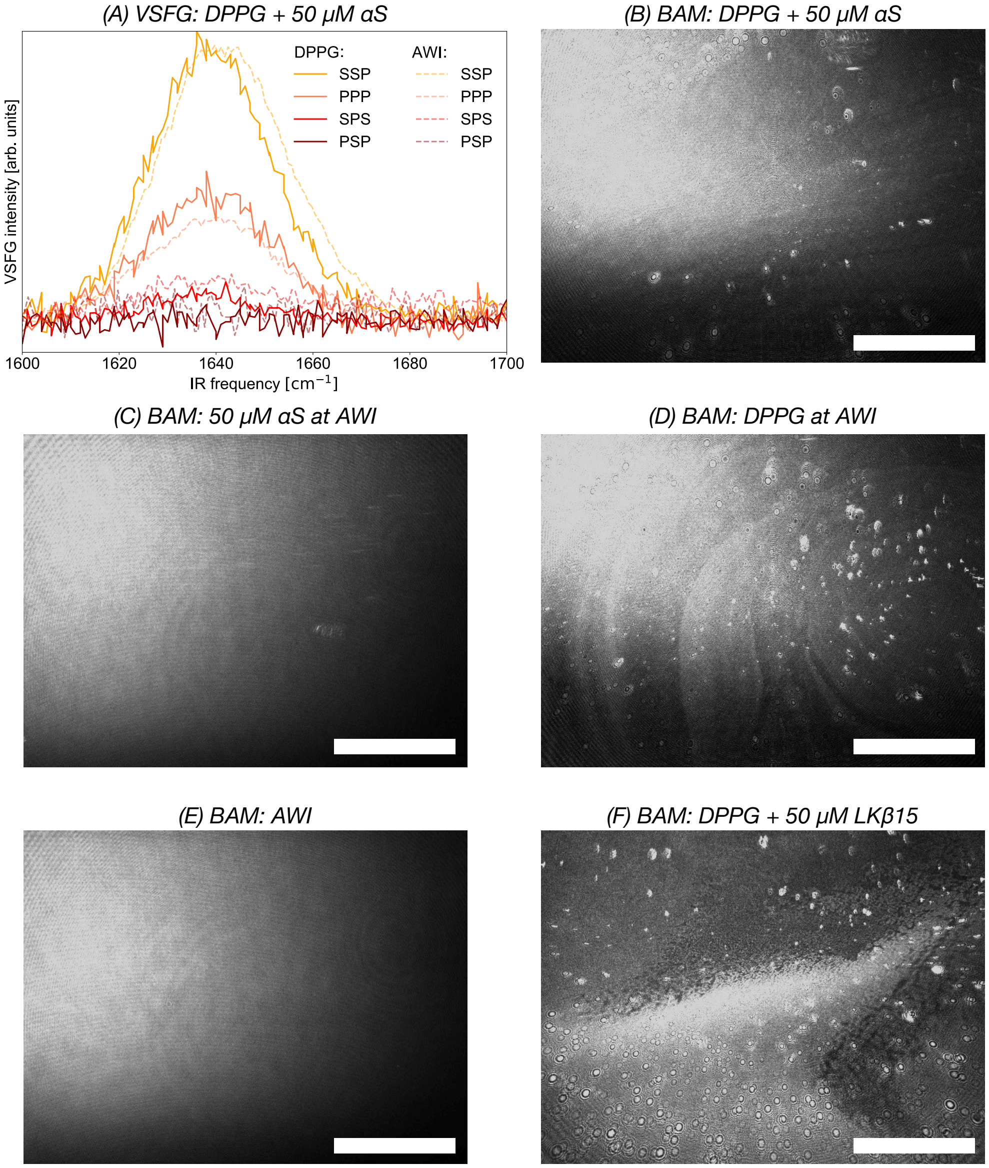
(A) VSFG: DPPG + 50 μM αS
VSFG intensity [arb. units]
IR frequency [cm⁻¹]
1600
1620
1640
1660
1680
1700
DPPG:
SSP
PPP
SPS
PSP
AWI:
SSP
PPP
SPS
PSP
(B) BAM: DPPG + 50 μM αS
(C) BAM: 50 μM αS at AWI
(D) BAM: DPPG at AWI
(E) BAM: AWI
(F) BAM: DPPG + 50 μM LKβ15

Supplementary Figure 14 *(preceding page)*: **(A) Comparison of the high-concentration VSFG spectra of $\alpha$S at the DPPG interface vs. at the air-water interface (AWI), and (B-F) additional experimental data – besides the AFM data – that shows that the spectral differences between the low- and high-$\alpha$S concentrations cannot be explained by lipid replacement at the AWI, using Brewster-angle microscopy (BAM).** The field of view = 3.65 x 2.74 mm). The best-matching ensembles of the two experimental VSFG datasets are significantly different.[29] BAM images of 50 $\mu$M $\alpha$S at the water-DPPG-air interface (B) and at the AWI (C) show differences, while the images look similar for pure DPPG monolayers (D) and 50 $\mu$M $\alpha$S at DPPG monolayers (B). We assign the $\sim$0.1 mm diameter spots to patches of DPPG, that are formed because we only dissolve the lipids in chloroform before dropcasting them on the water surface. As a positive control, we compare these results with BAM images of 50 $\mu$M LK$\beta$15 peptides (F), which can displace lipids at the AWI, and those images revealed large phase differences that were absent from the 50 $\mu$M $\alpha$S images (B). Taken together, the BAM images support the view – obtained from the VSFG and AFM data – that $\alpha$S does not replace the lipid monolayer at the AWI. Scale bars are 1 mm. Source data are provided in the Source Data file.

# Supplementary References

(1) Golbek, T. W.; Schmüser, L.; Rasmussen, M. H.; Poulsen, T. B.; Weidner, T. Lasalocid acid antibiotic at a membrane surface probed by sum frequency generation spectroscopy. *Langmuir* **2020**, *36*, 3184–3192.

(2) Wilhelm, B. G.; Mandad, S.; Truckenbrodt, S.; Kröhnert, K.; Schäfer, C.; Rammner, B.; Koo, S. J.; Claßen, G. A.; Krauss, M.; Haucke, V.; ; Urlaub, H.; Rizzoli, S. O. Composition of isolated synaptic boutons reveals the amounts of vesicle trafficking proteins. *Science* **2014**, *344*, 1023–1028.

(3) Borghi, R.; Marchese, R.; Negro, A.; Marinelli, L.; Forloni, G.; Zaccheo, D.; Abbruzzese, G.; Tabaton, M. Full length $\alpha$-synuclein is present in cerebrospinal fluid from Parkinson's disease and normal subjects. *Neuroscience letters* **2000**, *287*, 65–67.

(4) Seo, J.-H.; Rah, J.-C.; Choi, S. H.; Shin, J. K.; Min, K.; Kim, H.-S.; Park, C. H.; Kim, S.; Kim, E.-M.; Lee, S.-H., et al. $\alpha$-Synuclein regulates neuronal survival via Bcl-2 family expression and PI3/Akt kinase pathway. *The FASEB Journal* **2002**, *16*, 1–20.

(5) Bernal-Conde, L. D.; Ramos-Acevedo, R.; Reyes-Hernández, M. A.; Balbuena-Olvera, A. J.; Morales-Moreno, I. D.; Argüero-Sánchez, R.; Schüle, B.; Guerra-Crespo, M. Alpha-synuclein physiology and pathology: A perspective on cellular structures and organelles. *Frontiers in neuroscience* **2020**, *13*, 1399.

(6) Nowotarska, S. W.; Nowotarski, K. J.; Friedman, M.; Situ, C. Effect of structure on the interactions between five natural antimicrobial compounds and phospholipids of bacterial cell membrane on model monolayers. *Molecules* **2014**, *19*, 7497–7515.

(7) Gidalevitz, D.; Ishitsuka, Y.; Muresan, A. S.; Konovalov, O.; Waring, A. J.; Lehrer, R. I.; Lee, K. Y. C. Interaction of antimicrobial peptide protegrin with biomembranes. *Proceedings of the National Academy of Sciences* **2003**, *100*, 6302–6307.

(8) Jo, S.; Kim, T.; Iyer, V. G.; Im, W. CHARMM-GUI: a web-based graphical user interface for CHARMM. *Journal of computational chemistry* **2008**, *29*, 1859–1865.

(9) Rao, J. N.; Jao, C. C.; Hegde, B. G.; Langen, R.; Ulmer, T. S. A combinatorial NMR and EPR approach for evaluating the structural ensemble of partially folded proteins. *Journal of the American Chemical Society* **2010**, *132*, 8657–8668.

(10) Berendsen, H. J.; van der Spoel, D.; van Drunen, R. GROMACS: a message-passing parallel molecular dynamics implementation. *Computer physics communications* **1995**, *91*, 43–56.

(11) Piana, S.; Robustelli, P.; Tan, D.; Chen, S.; Shaw, D. E. Development of a force field for the simulation of single-chain proteins and protein–protein complexes. *Journal of chemical theory and computation* **2020**, *16*, 2494–2507.

(12) Jambeck, J. P.; Lyubartsev, A. P. Derivation and systematic validation of a refined all-atom force field for phosphatidylcholine lipids. *The journal of physical chemistry B* **2012**, *116*, 3164–3179.

(13) Jambeck, J. P.; Lyubartsev, A. P. Another piece of the membrane puzzle: extending slipids further. *Journal of chemical theory and computation* **2013**, *9*, 774–784.

(14) Grote, F.; Lyubartsev, A. P. Optimization of slipids force field parameters describing headgroups of phospholipids. *The Journal of Physical Chemistry B* **2020**, *124*, 8784–8793.

(15) Darden, T.; York, D.; Pedersen, L. Particle mesh Ewald: An N*log (N) method for Ewald sums in large systems. *The Journal of chemical physics* **1993**, *98*, 10089–10092.

(16) Bussi, G.; Donadio, D.; Parrinello, M. Canonical sampling through velocity rescaling. *The Journal of chemical physics* **2007**, *126*, 014101.

(17) Roeters, S. J.; van Dijk, C. N.; Torres-Knoop, A.; Backus, E. H. G.; Campen, R. K.; Bonn, M.; Woutersen, S. Determining In Situ Protein Conformation and Orientation from the Amide-I Sum-Frequency Generation Spectrum: Theory and Experiment. *The Journal of Physical Chemistry A* **2013**, *117*, 6311–6322, PMID: 23566310.

(18) Baiz, C. R. et al. Vibrational Spectroscopic Map, Vibrational Spectroscopy, and Intermolecular Interaction. *Chemical Reviews* **2020**, *120*, 7152–7218, PMID: 32598850.

(19) Zhuang, X.; Miranda, P. B.; Kim, D.; Shen, Y. R. Mapping molecular orientation and conformation at interfaces by surface nonlinear optics. *Phys. Rev. B* **1999**, *59*, 12632–12640.

(20) Hosseinpour, S.; Roeters, S. J.; Bonn, M.; Peukert, W.; Woutersen, S.; Weidner, T. Structure and dynamics of interfacial peptides and proteins from vibrational sum-frequency generation spectroscopy. *Chemical reviews* **2020**, *120*, 3420–3465.

(21) Roeters, S. J.; Golbek, T. W.; Bregnhøj, M.; Drace, T.; Alamdari, S.; Roseboom, W.; Kramer, G.; Šantl-Temkiv, T.; Finster, K.; Pfaendtner, J.; Woutersen, S.; Boesen, T.; Weidner, T. Ice-nucleating proteins are activated by low temperatures to control the structure of interfacial water. *Nature communications* **2021**, *12*, 1–9.

(22) Hale, G. M.; Querry, M. R. Optical Constants of Water in the 200-nm to 200-$\mu$m Wavelength Region. *Appl. Opt.* **1973**, *12*, 555–563.

(23) Ham, S.; Kim, J.-H.; Lee, H.; Cho, M. Correlation between electronic and molecular structure distortions and vibrational properties. II. Amide I modes of NMA–n D 2 O complexes. *The Journal of chemical physics* **2003**, *118*, 3491–3498.

(24) Lu, H.; Schäfer, A.; Lutz, H.; Roeters, S. J.; Lieberwirth, I.; Muñoz-Espí, R.; Hood, M. A.; Bonn, M.; Weidner, T. Peptide-controlled assembly of macroscopic calcium oxalate nanosheets. *The journal of physical chemistry letters* **2019**, *10*, 2170–2174.

(25) Weidner, T.; Castner, D. G. SFG analysis of surface bound proteins: a route towards structure determination. *Physical Chemistry Chemical Physics* **2013**, *15*, 12516–12524.

(26) Barth, A. Infrared spectroscopy of proteins. *Biochimica et Biophysica Acta (BBA)-Bioenergetics* **2007**, *1767*, 1073–1101.

(27) Kabsch, W.; Sander, C. Dictionary of protein secondary structure: pattern recognition of hydrogen-bonded and geometrical features. *Biopolymers: Original Research on Biomolecules* **1983**, *22*, 2577–2637.

(28) Jao, C. C.; Hegde, B. G.; Chen, J.; Haworth, I. S.; Langen, R. Structure of membrane-bound $\alpha$-synuclein from site-directed spin labeling and computational refinement. *Proceedings of the National Academy of Sciences* **2008**, *105*, 19666–19671.

(29) Strunge, K.; Burgin, T.; Golbek, T.; Roeters, S.; Pfaendtner, J.; Weidner, T. Umbrella-like helical structure of alpha-synuclein at the air-water interface observed with experimental and theoretical sum frequency generation spectroscopy. **2023**, DOI: 10.26434/chemrxiv–2023–lzc0.
