# Supplementary material for: Elevated concentrations cause upright alpha-synuclein conformation at lipid interfaces
Source: Nat Commun. 2023 Sep 18;14:5731. doi: 10.1038/s41467-023-39843-1 (PMC10507035; doi:10.1038/s41467-023-39843-1)
Supplement: Supplementary file 2 — Reporting Summary [file 41467_2023_39843_MOESM2_ESM.pdf]

nature portfolio

Corresponding author(s): Dr. Steven J. Roeters

Last updated by author(s): May 10, 2023

Reporting Summary

Nature Portfolio wishes to improve the reproducibility of the work that we publish. This form provides structure for consistency and transparency in reporting. For further information on Nature Portfolio policies, see our Editorial Policies and the Editorial Policy Checklist.

Statistics

For all statistical analyses, confirm that the following items are present in the figure legend, table legend, main text, or Methods section.

n/a	Confirmed	
x		The exact sample size (*n*) for each experimental group/condition, given as a discrete number and unit of measurement
	x	A statement on whether measurements were taken from distinct samples or whether the same sample was measured repeatedly
x		The statistical test(s) used AND whether they are one- or two-sided<br>*Only common tests should be described solely by name; describe more complex techniques in the Methods section.*
	x	A description of all covariates tested
x		A description of any assumptions or corrections, such as tests of normality and adjustment for multiple comparisons
	x	A full description of the statistical parameters including central tendency (e.g. means) or other basic estimates (e.g. regression coefficient) AND variation (e.g. standard deviation) or associated estimates of uncertainty (e.g. confidence intervals)
x		For null hypothesis testing, the test statistic (e.g. *F*, *t*, *r*) with confidence intervals, effect sizes, degrees of freedom and *P* value noted<br>*Give P values as exact values whenever suitable.*
x		For Bayesian analysis, information on the choice of priors and Markov chain Monte Carlo settings
x		For hierarchical and complex designs, identification of the appropriate level for tests and full reporting of outcomes
x		Estimates of effect sizes (e.g. Cohen's *d*, Pearson's *r*), indicating how they were calculated

Our web collection on statistics for biologists contains articles on many of the points above.

Software and code

Policy information about availability of computer code

Data collection	"Andor SOLIS for Spectroscopy" (the manufacturer's software that operates the CCD camera) version 4.30.30034.0 and for the out-of-equilibrium MD simulation the CHARMM-GUI webserver and GROMACS v2021.4 were used.
Data analysis	The raw experimental data was processed with home-written python script (described in the SI) . The spectral calculations were performed with an f90 script that has been previously described in J. Phys. Chem. Lett. 2019, 10, 9, 2170–2174, Langmuir 2020, 36, 40, 11855–11865, and J. Phys. Chem. Lett. 2022, 13, 22, 5025–5029. The frame selection was finally performed in a second home-written python script. As referred to in the Code Availability section, all of the home-written scripts are available upon reasonable request from the authors and from the Visca github page.

For manuscripts utilizing custom algorithms or software that are central to the research but not yet described in published literature, software must be made available to editors and reviewers. We strongly encourage code deposition in a community repository (e.g. GitHub). See the Nature Portfolio guidelines for submitting code & software for further information.

Data

Policy information about availability of data

All manuscripts must include a data availability statement. This statement should provide the following information, where applicable:

- Accession codes, unique identifiers, or web links for publicly available datasets
- A description of any restrictions on data availability
- For clinical datasets or third party data, please ensure that the statement adheres to our policy

The datasets generated during and/or analyzed during the current study are available from the corresponding author upon reasonable request, in the Source Data file, and on Zenodo (https://zenodo.org/record/7916728#.ZFrQ3OxBw-Q).

Human research participants

Policy information about studies involving human research participants and Sex and Gender in Research.

Reporting on sex and gender	N/A
Population characteristics	N/A
Recruitment	N/A
Ethics oversight	N/A

Note that full information on the approval of the study protocol must also be provided in the manuscript.

Field-specific reporting

Please select the one below that is the best fit for your research. If you are not sure, read the appropriate sections before making your selection.

[x] Life sciences [ ] Behavioural & social sciences [ ] Ecological, evolutionary & environmental sciences

For a reference copy of the document with all sections, see nature.com/documents/nr-reporting-summary-flat.pdf

Life sciences study design

All studies must disclose on these points even when the disclosure is negative.

Sample size	N/A (the sample size is not a relevant parameter in these in vitro chemical-physics vibrational spectroscopy experiments. In this field several in vitro experiments are performed, and when they reproduce well, we continue the research by analyzing the datasets in a structural manner).
Data exclusions	There was no data excluded.
Replication	All experiments were were performed independently twice, and successfully reproduced. The derived structural ensembles were also reproduced between reproductions; see details in the Methods section of the article.
Randomization	N/A (see "Sample size" answer - also randomization is not a relevant parameter in such in vitro experiments.)
Blinding	N/A (all experiments were performed and analyzed by the main authors, so it was impossible to blind our samples without loosing track of which dataset belongs to which sample. However, as all code and experimental data has been made accessible, everyone interested will be able to reproduce these results).

Reporting for specific materials, systems and methods

We require information from authors about some types of materials, experimental systems and methods used in many studies. Here, indicate whether each material, system or method listed is relevant to your study. If you are not sure if a list item applies to your research, read the appropriate section before selecting a response.

Materials & experimental systems

n/a	Involved in the study
☒	☐ Antibodies
☒	☐ Eukaryotic cell lines
☒	☐ Palaeontology and archaeology
☒	☐ Animals and other organisms
☒	☐ Clinical data
☒	☐ Dual use research of concern

Methods

n/a	Involved in the study
☒	☐ ChIP-seq
☒	☐ Flow cytometry
☒	☐ MRI-based neuroimaging
